# Volcano-type relationship between oxidation states and catalytic activity of single-atom catalysts towards hydrogen evolution

**DOI:** 10.1038/s41467-022-33589-y

**Published:** 2022-10-04

**Authors:** Dong Cao, Haoxiang Xu, Hongliang Li, Chen Feng, Jie Zeng, Daojian Cheng

**Affiliations:** 1 State Key Laboratory of Organic-Inorganic Composites and Beijing Advanced Innovation Center for Soft Matter Science and Engineering, Beijing University of Chemical Technology, Beijing, 100029 PR China; 2 Hefei National Laboratory for Physical Sciences at the Microscale, Key Laboratory of Strongly-Coupled Quantum Matter Physics of Chinese Academy of Sciences, Key Laboratory of Surface and Interface Chemistry and Energy Catalysis of Anhui Higher Education Institutes, Department of Chemical Physics, University of Science and Technology of China, Hefei, Anhui 230026 PR China

**Keywords:** Electrocatalysis, Electrocatalysis, Nanoscale materials

## Abstract

To date, the effect of oxidation state on activity remains controversial in whether higher or lower oxidation states benefit the enhancement of catalytic activity. Herein, we discover a volcanic relationship between oxidation state and hydrogen evolution reaction activity based on Os single-atom catalysts. Firstly, a series of Os SACs with oxidation states ranging from  + 0.9 to  + 2.9 are synthesized via modifying the coordination environments, including Os-N_3_S_1_, Os-N_4_, Os-S_6_, Os-C_3_, and Os-C_4_S_2_. A volcano-type relation between oxidation states and hydrogen evolution activity emerge with a summit at a moderate experimental oxidation state of  + 1.3 (Os-N_3_S_1_). Mechanism studies illustrate that with increasing oxidation states, the adsorption of H atoms on Os is strengthened due to increased energy level and decreased occupancy of anti-bonding states of Os-H bond until the anti-bonding states become empty. Further increasing the oxidation states weakens hydrogen adsorption because of the decreased occupancy of Os-H bonding states. In this work, we emphasize the essential role of oxidation state in manipulating activity, which offers insightful guidance for the rational design of single-atom catalysts.

## Abstract (web summary)

While single atom catalysis offers high efficiency for materials use, different possible atomic configurations yield differing activities. Here, authors modulate single-atom Os coordinations to show a volcano relationship between oxidation state and H_2_ evolution electrocatalytic activities.

## Introduction

Single-atom catalysts (SACs) have received growing interests in various catalytic fields due to the utmost utilization efficiency of metal atoms^1–11^. In addition, distinct local structure of SACs also provides an in-depth comprehension to the intrinsic structure-performance relationship during catalytic process^12–20^. Notably, when metal single atoms are anchored on the supports, the metal-support interaction and the heterogeneity of the supports endow single atoms with tunable electronic properties, such as oxidation state, highest occupied molecular orbital (HOMO), lowest unoccupied molecular orbital (LUMO), spin states, etc., resulting in controllable catalytic properties^21–25^. For instance, Rh atom doped rutile VO_2_ exhibited higher NH_3_BH_3_ hydrolysis activity than Rh doped monoclinic VO_2_ due to the higher occupied state of single Rh atoms supported on rutile VO_2_^21^. Mn-C_3_N_4_ showed high solar-driven water splitting activity, which was mainly dependent on its spin state with an optimized *e*_g_ occupancy (≈0.95)^26^.

Among these electronic properties, oxidation state is considered as a typical parameter to correlate with the catalytic activity. However, specific relationship between oxidation state and catalytic activity remains controversial in whether higher or lower oxidation states benefit the enhancement of catalytic activity for SACs. For example, reported metallic Pt SACs presented higher activities than highly oxidized Pt SACs during CO oxidation because the adsorption strengths of CO increased as the Pt atom became more metallic^27^. Similarly, Pt_1_/Fe_2_O_3_ SACs with lower oxidation state exhibited more excellent 3-nitrostyrene hydrogenation activity than other Pt_1_/Fe_2_O_3_ SACs with higher oxidation states because lower oxidation state was more beneficial for H_2_ adsorption and dissociation^28^. On contrary, Fe-N_4_ SACs with the highest oxidation state displayed higher benzene oxidation reaction activity compared to Fe-N_3_C and Fe-N_2_C_2_ SACs because Fe-N_4_ facilitated the generation and activation of the vital intermediate O= Fe=O species, enhancing the ability to activate C-H bond^29^. Considering the controversial role played by oxidation state, it is necessary to clarify the specific role and demonstrate the underlying catalytic mechanism.

In this work, we select osmium (Os) as research object to fully discuss the relationship between oxidation state and catalytic activity due to its rich valence states (−2 to +8). Firstly, we successfully synthesized atomically dispersed Os sites with different oxidation states (from  + 0.9 to  + 2.9) by changing coordination environments, including Os-N_3_S_1_, Os-N_4_, Os-S_6_, Os-C_3_, and Os-C_4_S_2_. It was found that a volcanic curve was obtained between oxidation state and hydrogen evolution reaction (HER) activity based on various Os SACs. Specifically, Os-N_3_S_1_ SACs with a moderate experimental oxidation state ( + 1.3) displayed the best acidic HER performance compared to other SACs, which only needed 22 mV at 10 mA cm^−2^. Further mechanism studies disclosed that with increasing oxidation states (decreasing valence electron numbers), the adsorption of H atoms on Os was strengthened due to the increased energy level and decreased occupancy of anti-bonding states of Os-H bond until the anti-bonding states become empty. Further increasing the oxidation states weakened hydrogen adsorption because of the decreased occupancy of Os-H bonding states near the Fermi level. This work provided Os SACs for HER and revealed the essential role of oxidation state in manipulating activity, which offers essential guidance for the rational design of SACs.

## Results

### Synthesis and characterizations of Os SACs

**Fig. 1:**
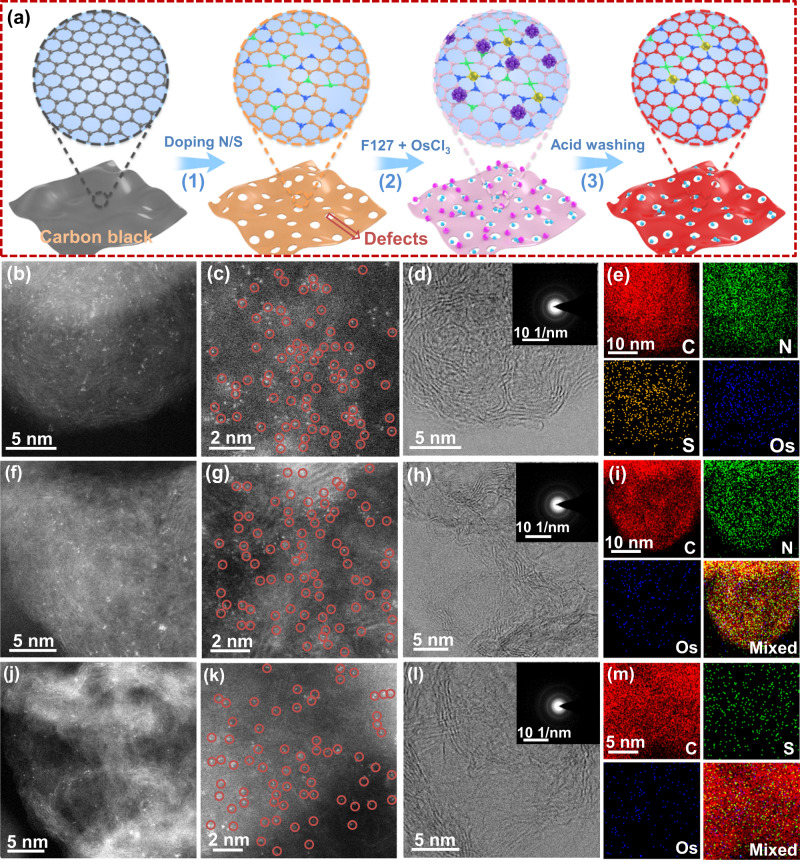
Synthesis strategy and electron microscopic characterization. **a** Synthesis route of Os atomically dispersed catalysts. (1) The introduction of N and S atoms. (2) Atomically dispersed Os are supported on the carrier. (3) Acid washing step. **b**, **c** Aberration corrected HAADF-STEM images of Os/CNS in different scale bars. **d** Aberration corrected TEM image of Os/CNS. The inset corresponds to the SAED pattern of the sample. **e** Corresponding EDS elemental mapping of Os/CNS. **f**, **g** Aberration corrected HAADF-STEM images of Os/CN in different scale bars. **h** Aberration corrected TEM image of Os/CN. The inset corresponds to the SAED pattern of the sample. **i** Corresponding EDS elemental mapping of Os/CN. **j**, **k** Aberration corrected HAADF-STEM images of Os/CS in different scale bars. **l** Aberration corrected TEM image of Os/CS. The inset corresponds to the SAED pattern of the sample. **m** Corresponding EDS elemental mapping of Os/CS.

To fully discuss the relationship between oxidation state and catalytic activity, we synthesized atomically dispersed Os catalysts with controllable coordination environments and verified the proposed regulation mechanism of coordination configuration to optimize HER catalytic activity. The synthetic strategy of atomically dispersed Os sites supported on N and S co-doped carbon materials (Os/CNS) is shown in Fig. 1a. First, N and S atoms were introduced into carbon black by adopting urea and 2,2’-bithienyl as the nitrogen and sulfur sources, respectively. After that, Pluronic F127 was fully mixed with CNS support in 30 mL of deionized water. Then, osmium trichloride (OsCl_3_) solution was slowly added into above suspension. After stirring for 6 h, the mixture was centrifuged and dried in a vacuum drying oven. Finally, the dried products were calcined in a tubular furnace at 400 °C and atomically dispersed Os/CNS sample was successfully fabricated after washing with acid solution for 12 h. Moreover, other atomically dispersed Os single atoms supported on different carriers, such as pure carbon black (C), N doped graphite carbon (CN), and S doped graphite carbon (CS), were also prepared by changing the doping heteroatom in carbon support while keeping other conditions unchanged.

To clearly obtain the atomic information of these catalysts, aberration-corrected high-angle annular dark-field scanning transmission electron microscopy (AC-HAADF-STEM) was employed^30–32^. Figure 1b, c exhibited the AC-HAADF-STEM images of Os/CNS in different scale bars. It is clear that atomically dispersed Os sites were uniformly anchored on the CNS support. Subsequently, aberration-corrected TEM image of Os/CNS was shown in Fig. 1d. It is obvious that no large nanoparticles were observed. The evident lattice stripe of CNS support revealed its good crystallinity, which indicated a superior electronic conductivity. The inset of Fig. 1d corresponded to the selected area electron diffraction (SAED) of Os/CNS sample. No diffraction spots further disclosed the absence of Os particles. Then, Fig. 1e directly displayed the homogeneous distribution of C, N, S, and Os elements in Os/CNS sample. In addition, to regulate the coordination structure of Os active sites, we changed the CNS into CN support while kept other synthesis conditions remained. Similarly, Os/CN displayed a uniform distribution of isolated Os atoms on the CN carrier (Fig. 1f, g). The aberration-corrected TEM image and SAED pattern verified the polycrystalline characteristics of the CN carrier (Fig. 1h). Meanwhile, the C, N, and Os elements were also uniformly dispersed across the elemental mapping (Fig. 1i). Moreover, as a control sample, Os single atom supported on CS carrier was also successfully synthesized. Obviously, Fig. 1j, k clearly revealed the even dispersion of Os atoms in Os/CS. The aberration-corrected TEM image of Os/CS also confirmed the uniform distribution of Os single atoms on CS carrier (Fig. 1l). The SAED pattern of Os/CS sample was the same as those of Os/CNS and Os/CN (inset in Fig. 1l). Afterwards, Fig. 1m exhibited the uniform distribution of Os, C, and S atoms for Os/CS. To fully discuss the influence of coordination environments, we also fabricated atomically dispersed Os/CS-2 and Os/C catalysts (Supplementary Figs. 1a–d and 2a–e). Furthermore, elemental mapping tests of Os/CS-2 and Os/C suggested the homogeneous distribution of corresponding elements on entire structure (Supplementary Figs. 3d, 4e). Additionally, the low-resolution STEM images of Os/CNS, Os/CN, Os/CS, and Os/CS-2 were characterized (Supplementary Fig. 3a–d). The basic characterizations of pure carbon-based carriers were also completed (Supplementary Figs. 4–11). Besides, Os nanoparticles (NPs) were also synthesized for comparison (Supplementary Fig. 12a, b).

**Fig. 2:**
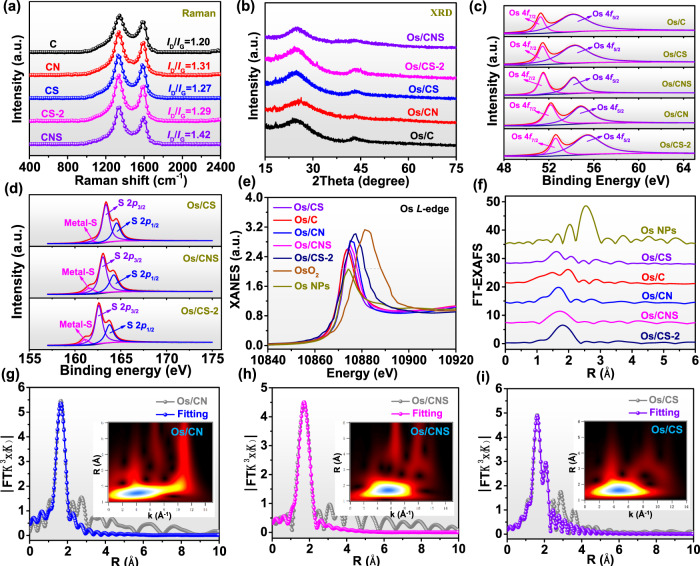
Spectral characterization. **a** Raman spectra of different samples. **b** XRD patterns. **c**, **d** High-resolution XPS spectra of Os 4 *f* and S 2*p*, respectively. **e** XANES of Os *L*-edge for different samples. **f** FT-EXAFS curves of every sample. **g**–**i** FT-EXAFS fitting curves of Os/CN, Os/CNS, and Os/CS SACs, respectively. The insets in (**g**–**i**) correspond to WT-EXAFS of Os/CN, Os/CNS, and Os/CS, respectively.

As shown in Fig. 2a, it could be found from Raman spectra that the values of *I*_D_/*I*_G_ changed from 1.20 for C carrier to 1.31, 1.27, 1.29, and 1.42 for CN, CS, CS-2, and CNS carriers, respectively. Thus, the surface defects obviously increased after doping N and S atoms into carbon black due to the enlarged values of *I*_D_/*I*_G_ (Fig. 2a). The increased level of defects could contribute to the anchoring of metal atoms. According to the inductively coupled plasma-optical emission spectrometer (ICP-OES) test, the mass loading of Os for Os/C, Os/CN, Os/CNS, Os/CS, and Os/CS-2 was 3.17%, 4.56%, 4.61%, 3.80%, and 4.03%, respectively. Then, to investigate more information of Os single atom supported on different carbon materials, X-ray diffraction (XRD) and N_2_ adsorption-desorption isotherms tests were applied. The XRD results of different Os SACs were almost the same as that of pure carbon black, disclosing no corresponding diffraction peak of Os nanoparticles (Fig. 2b and Supplementary Fig. 13). The XRD results of different SACs were consistent with SAED, suggesting the successful preparation of various Os SACs. In addition, the nitrogen adsorption-desorption isotherm of different samples demonstrated that the specific surface areas of Os/C, Os/CN, Os/CNS, Os/CS, and Os/CS-2 were 1418.5, 1389.3, 1370.3, 1382.7, and 1325.9 m^2^/g, respectively (Supplementary Fig. 14a–e). The larger surface area exposed more active centers for water splitting and contributed to the adsorption and desorption of reactants and products.

X-ray photoelectron spectroscopy (XPS) was adopted to unveil the chemical composition and structure of each element in single atom samples^33^. In the Os 4 *f* XPS spectra, the Os 4*f*_7/2_ and Os 4*f*_5/2_ peaks of Os/C were at 51.2 and 54.1 eV, respectively, which were higher than those (50.3 and 53.1 eV, respectively) of Os nanoparticles (Fig. 2c and Supplementary Fig. 15). The oxidation state of Os nanoparticles is nearly 0. Then, the binding energies of Os in Os/CS (51.4 and 54.3 eV) were moved to larger values compared to Os/C sample (Fig. 2c). For Os/CNS, XPS survey spectrum firstly revealed the co-existence of Os, N, S, and C elements (Supplementary Fig. 16). Then, the two characteristic peaks of Os 4*f*_7/2_ and Os 4*f*_5/2_ were obtained at 51.6 and 54.2 eV, respectively (Fig. 2c). Additionally, the binding energies of Os in Os/CN were located at 52.2 and 54.8 eV. The locations of Os 4*f*_7/2_ and Os 4*f*_5/2_ for Os/CS-2 were centered at 52.6 and 55.5 eV, respectively. Thus, the oxidation state of different Os SACs increased in the order of Os/C < Os/CS < Os/CNS < Os/CN < Os/CS-2 because of the positive movement of corresponding binding energies, suggesting that doped heteroatoms effectively regulated the oxidation state of Os sites. Moreover, Fig. 2d displayed the S 2*p* spectra of Os/CS, Os/CNS, and Os/CS-2. Importantly, the S 2*p* curve of Os/CNS can be divided into three peaks at 161.6 eV (Metal-S bond), 163.0 eV (C-S-C), and 164.2 eV (C-S-C). In addition, S 2*p* XPS spectra of Os/CS and Os/CS-2 also exhibited the same three peaks. The XPS spectrum of S 2*p* for pure CNS revealed no Metal-S bonds (Supplementary Fig. 17). Thus, it can be inferred that Os in Os/CNS, Os/CS, and Os/CS-2 directly bonded with S atom, suggesting different coordination environments of these three samples from the traditional metal-N_4_ structures. In addition, the N 1 *s* spectra of Os/CNS and Os/CN displayed four peaks at 398.5, 399.5, 400.8, and 401.6 eV, corresponding to pyridinic N, Metal coordinated N, pyrrolic N, and graphitic N, respectively (Supplementary Fig. 18). The successful doping of different heteroatoms precisely regulated the oxidation states of active centers, which should change the catalytic performance of atomically dispersed catalysts.

X-ray absorption fine structure (XAFS) analyses^34,35^, including X-ray absorption near-edge structure (XANES) and extended X-ray absorption fine structure (EXAFS), were conducted to detect the specific coordination environments of Os SACs. The Os *L*-edge XANES spectra of Os SACs, OsO_2_, and Os nanoparticles were shown in Fig. 2e. The oxidation states of catalysts estimated from the integrated area under the white-line intensity of Os *L*-edge was displayed in Supplementary Fig. 19. The oxidation state of Os single atoms increased in the order of Os/C ( + 0.9) <Os/CS ( + 1.1) <Os/CNS ( + 1.3) <Os/CN ( + 1.9) <Os/CS-2 ( + 2.9), revealing that doped heteroatoms effectively regulated the oxidation state of Os sites (Supplementary Fig. 19). It was consistent with the analyses of XPS results. Furthermore, EXAFS results were collected to further acquire precise coordination configuration, including coordination numbers and bond lengths (Fig. 2f). The Fourier-transformed (FT) k^3^-weighted EXAFS spectrum of Os/CN SAC exhibited a prominent peak at 1.68 Å, which was attributed to the Os-N bonds (Fig. 2f). Moreover, the first peak for Os/CNS was wider and moved positively compared to Os/CN, implying changes in coordination structure due to the doping of S atoms. Meanwhile, as displayed in Fig. 2f, the coordination environments of Os/CS-2, Os/CS, and Os/C were different from Os/CN with different shaped FT k^3^-weighted EXAFS spectra. Besides, Fig. 2f further demonstrated no obvious Os-Os bonds in Os/CNS, Os/CS, Os/CN, Os/CS-2, and Os/C compared to Os NPs. Afterwards, quantitative EXAFS fitting analyses of different Os SACs and Os NPs were used to confirm the detailed local coordination environments (Fig. 2g–i and Supplementary Fig. 20a–c). The corresponding fitting parameters were listed in Supplementary Table 1. Notably, Fig. 2g manifested that the main peak at 1.68 Å for Os/CN could be satisfactorily interpreted as Os-N first shell coordination and the corresponding coordination number was 4 with a bonding distance of 2.04 Å (Supplementary Table 1). Moreover, Os/CNS sample included Os-S and Os-N bonds in the first shell coordination (Fig. 2h) and the specific coordination numbers of Os-N and Os-S bonds were 3 and 1, respectively, implying that Os-N_3_S_1_ coordination structure was successfully constructed (Supplementary Table 1). The introduce of S atoms could effectively change the dispersion of electrons around active sites, resulting in precise regulation of oxidation state. Obviously, the best-fitting analysis of Os/CS further revealed the first shell coordination of Os-C at 1.65 Å and Os-S at 2.05 Å with a coordination number of 4 and 2, respectively (Fig. 2i and Supplementary Table 1). In addition, Os-S_6_ and Os-C_3_ coordination configurations can also be synthesized when Os is supported on CS-2 and pure C, respectively (Supplementary Fig. 20d–f). To further distinguish the formation of Os-C/N coordination in Os/CN and Os/CNS, the soft X-ray absorption spectroscopy at N *K*-edge and C *K*-edge of catalysts was applied. Based on C *K*-edge spectra for Os/CN and Os/CNS, the peak C1 (285.7 eV) was derived from C-C π* (ring) excitations, the peak C2 (288.8 eV) was originated from C-N-C, and peak C3 (293.0 eV) corresponded to C-C δ* (ring), respectively (Supplementary Fig. 21). Importantly, C1, C2, and C3 peaks of Os/CN and Os/CNS were almost similar as that of pure carrier, indicating Os atoms did not coordinate with C atoms in carrier. In addition, N *K*-edge spectra for Os/CN and Os/CNS disclosed that four typical peaks (N1, N2, N3, and N4) are directly observed, which corresponded to pyridinic N, Metal coordinated N, pyrrolic N, and graphitic N species, respectively (Supplementary Fig. 22). Therefore, it further confirmed the Os atoms bonded with N atoms in Os/CN and Os/CNS, which was consistent with the analyses of XPS results. Thus, Os/CS-2, Os/CN, Os/CNS, Os/CS, and Os/C can also be regarded as Os-S_6_, Os-N_4_, Os-N_3_S_1_, Os-C_4_S_2_ and Os-C_3_ SACs in this study, respectively. Five different coordinated structure catalysts were precisely constructed in this work, which could be used to disclose the relationship between coordination environments and catalytic activity. Additionally, wavelet transform (WT) was further conducted to disclose the Os *L*-edge EXAFS oscillations of different Os SACs (insets in Fig. 2g–i and Supplementary Fig. 20). It is obvious that WT analyses of Os-N_4_, Os-N_3_S_1_, and Os-C_4_S_2_ exhibited the maximum intensity at approximately 4.0, 5.8, and 5.0 Å^−1^, respectively, which were quite different from the Os nanoparticles (13 Å^−1^, Supplementary Fig. 20f). Meanwhile, Os-C_3_ and Os-S_6_ also displayed the maximum intensity at nearly 4.4 and 5.9 Å^−1^, respectively, which were different from Os-N_4_ and Os-C_4_S_2_. Therefore, Os sites with different coordination environments were confirmed as atomically distributed in this work.

### Electrochemical performance of Os SACs for HER

**Fig. 3:**
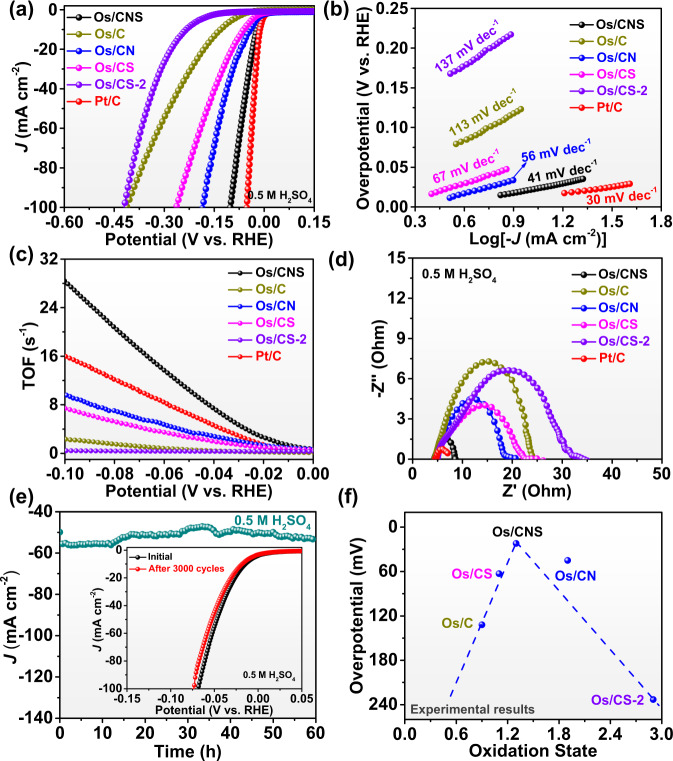
Electrochemical tests. **a** HER polarization curves in acidic media. **b** Tafel plots in acidic medium. **c** TOF plots in acid. **d** Nyquist plots in acidic media. **e** The chronoamperometric curves of Os/CNS in acidic media (The inset corresponds to polarization curves measured in 0.5 M H_2_SO_4_ before and after 3000 CV cycles). **f** The relationship between oxidation state and overpotential obtained from experiments for different Os SACs.

To disclose the influence of coordination structure on catalytic performance, we measured the electrochemical HER activities of Os catalysts in 0.5 M H_2_SO_4_^36,37^. According to the linear sweep voltammetry (LSV) curves, Os/CNS, Os/C, Os/CN, Os/CS, Os/CS-2, and 20% Pt/C required overpotentials of 22, 132, 45, 63, 233, and 13 mV to get a current density of 10 mA cm^−2^ (Fig. 3a). The onset potentials (at current density of 1 mA cm^−2^) for Os/CNS, Os/C, Os/CN, Os/CS, Os/CS-2, and 20% Pt/C corresponded to 5, 47, 11, 18, 129, and 3 mV, respectively^38^. Therefore, the HER activities of different Os SACs in this work were as follows: Os/CNS > Os/CN > Os/CS > Os/C > Os/CS-2. Moreover, the LSV curves in acidic media identified that the doping of heteroatoms (N and S) effectively regulated the HER performance of SACs. Meanwhile, Os sites served as the main active centers for HER while carbon supports stabilized the metal atoms because pure carbon materials showed poor performance for HER in acidic media (Supplementary Fig. 23). In addition, the fitting Tafel slopes in acidic medium were illustrated in Fig. 3b. The Tafel slopes of Os/CNS, Os/C, Os/CN, Os/CS, Os/CS-2, and 20% Pt/C were 41, 113, 56, 67, 137, and 30 mV dec^−1^, respectively, interpreting the fastest HER kinetics of the Os-N_3_S_1_ among single atom samples with different coordination structures^39,40^. Importantly, Os/CNS, Os/C, Os/CN, Os/CS, and Os/CS-2 involved in Volmer-Heyrovsky mechanism. Meanwhile, at overpotential of 50 mV, the turnover frequency (TOF) values of Os/CNS, Pt/C, Os/C, Os/CN, Os/CS, and Os/CS-2 were 10.55, 6.49, 0.62, 3.60, 2.66, and 0.34 s^−1^, respectively (Fig. 3c). TOF plots further revealed that Os/CNS illustrated highest intrinsic catalytic activity for HER in acidic medium because it displayed the largest TOF values under the same potentials in comparison with other SACs in this work (Fig. 3c). Moreover, Os/CNS also showed better HER activity compared to some reported catalysts (Supplementary Table 2). Additionally, electrochemical impedance spectroscopy (EIS) measurements were employed to provide further insight into the electrical conductivity of SACs in acidic media^41^. As illustrated in Fig. 3d, Os/CNS displayed the smallest value of charge transfer resistance (*R*_ct_) in acidic medium compared to other SACs, indicating its fastest HER kinetics. Then, the electrochemical double-layer capacitance (*C*_dl_) was also used to estimate the electrochemically active surface area of electrocatalysts (Supplementary Figs. 24a–f, 25). Specially, the *C*_dl_ value (29.2 mF cm^−2^) of Os/CNS was larger than that of other SACs, suggesting that it exhibited larger electrochemically-active surface area (ECSA) because ECSA is positively correlated with *C*_dl_^42^. Moreover, stability is also an important parameter to access the HER performance of electrocatalysts. The long-term durability measurements of Os/CNS sample, including chronoamperometric curves and accelerated linear potential sweeps, were performed in 0.5 M H_2_SO_4_ solution. It is obvious that the current density of Os/CNS sample displayed negligible decrease after more than 50 h test (Fig. 3e). Moreover, LSV curves of Os/CNS also declined negligibly in acidic condition after 3000 cycles (inset of Fig. 3e). Os/C, Os/CN, Os/CS, and Os/CS-2 also performed excellent stability, as shown in Supplementary Fig. 26a–d. Furthermore, Supplementary Fig. 27a–d manifested that Os/CNS showed excellent structural stability due to the uniform distribution of Os atoms on carrier after HER. In Fig. 3f, oxidation states analyzed from experiments were used to correlate with HER overpotential in acidic medium. It was found that different coordination environments induced various oxidation states of Os single-atom, which directly affected catalytic performance in a volcanic curve relation. Obviously, Os/CNS showed highly active catalytic activity owing to its moderate oxidation state (+1.3). Experimental discovery verified that the oxidation state of isolated metal atom illustrated a volcanic curve with HER activity. The fundamental understanding on the role of oxidation state in coordination-dependent activity offers valuable guidance for a rational design of SACs through coordination environment engineering.

### Theoretical studies between oxidation state and HER activity for Os SACs

According to the local coordination environments of graphene-supported metal SACs reported by previous works^43,44^, density functional theory (DFT) calculations were employed to explore the origin of coordination-dependent catalytic activity among Os SACs. To further precisely identify the local coordination environment of each Os SACs, we constructed all possible configuration according to quantitative EXAFS fitting analyses, as shown in Supplementary Fig. 28a–k. Moreover, we also performed DFT calculations to explore thermodynamics preference of bonds formation between Os-C and Os-N in Os/CN and Os/CNS, according to energy change of the process (Supplementary Fig. 29a, b). As shown in Supplementary Fig. 29a, as for Os/CNS with N source during synthesis, the endothermic process (positive value of energy change) for Os single atom, from forming Os-N_3_S_1_ to Os-C_1_N_2_S_1_ or Os-C_2_N_1_S_1_ local coordination, indicating that Os single atoms tended to form Os-N bond without Os-C coordination. Likewise, as for Os/CN with N source during synthesis, Os single atoms tended to form Os-N_4_ rather than Os-C_2_N_2_ or Os-C_4_ local coordination (Supplementary Fig. 29b). Thus, we did not consider candidate configuration with Os-C bonds in Os/CN and Os/CNS. The bond lengths between Os and nearest neighbor were summarized in Supplementary Table 3, which can be compared with experimental EXAFS results (Supplementary Table 1). The oxidation states of Os single-atom were estimated for all possible models by normalizing valence electron number (by Bader charges analysis) to those of bulk compounds (Os, Os_3_O, and OsO_2_) with known oxidation states (Supplementary Fig. 30). The results were shown in Supplementary Table 3, which could confirm whether it was in good agreement with the relative order derived from XPS and XANES analyses. We also checked the stability of the proposed possible configurations of Os SACs against metal atom aggregation, in order to confirm that the proposed possible catalysts are experimentally feasible (Supplementary Fig. 31a, b and supplementary method). All configurations meeting oxidation state, bond length and stability against sintering were labeled in red color in Supplementary Table 3, which were selected for the further investigations on HER theoretical activity.

**Fig. 4:**
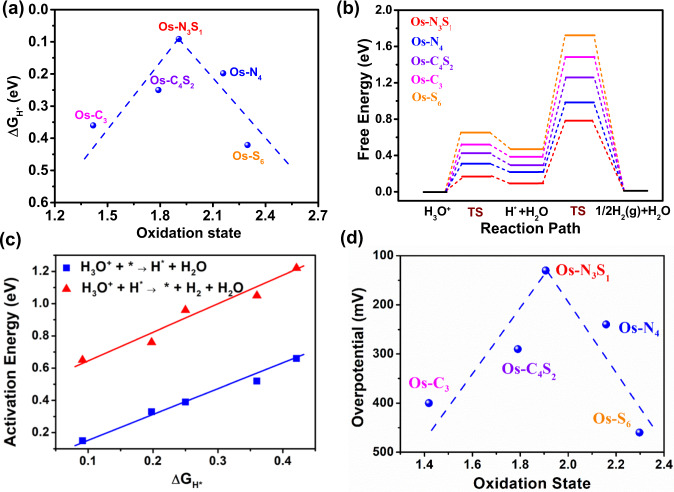
DFT calculations. **a** ΔG_H*_ as a function of oxidation state of Os SACs. **b** Gibbs free energy diagram of HER in acid media on Os SACs. **c** Energy barrier of elementary steps in acidic media as a function of free adsorption energy of atomic hydrogen (ΔG_H*_). **d** Theoretical overpotential (at 10 mA/cm^2^) as a function of oxidation state for different Os SACs.

The adsorption free energy of hydrogen, Δ*G*_H*_, has been widely considered as a useful descriptor for acidic HER catalytic activity^45^. In order to explore whether oxidation state acts as a reliable electronic structure-involved descriptor, the relationship between oxidation state of Os single-atoms and Δ*G*_H*_ for the corresponding Os SACs was built. It was found that diverse coordination environments of Os SACs brought about distinguishable oxidation states (Fig. 4a). It suggested that the electronic hybridization between center single-atom and proximal atoms from the support modified the valence electron occupation through electronic metal-support interaction (EMSI)^46^. Among all considered Os SACs, their Δ*G*_H*_ were above zero. For Os SACs with stronger binding strength of H, H^+^ can be more easily captured from the electrolyte and combined with adsorbed H* to form gaseous H_2_. What’s more, a volcanic correlation existed between oxidation state and ΔG_H*_. Since Os-N_3_S_1_ possessed the moderate Os oxidation state of  + 1.9, it displayed the strongest binding strength of H and might accordingly show better acidic HER performance than other Os SACs with distinct Os oxidation states. Figure 4a suggested that the activity of Os SACs for acidic HER might be controlled by oxidation state as it adjusted Δ*G*_H*_. Hence, oxidation state has potential to be a reliable descriptor to predict acidic HER activity of Os SACs.

DFT calculations were further employed to explore the role of oxidation state in coordination-dependent catalytic kinetics among Os SACs. According to the characteristic of Tafel slope in Fig.3b, we inferred that Os SACs may prefer Volmer-Heyrovsky mechanism. As for the Volmer-Heyrovsky mechanism in acidic HER, the reaction pathway could be described as a three-state diagram consisting of an initial free H_3_O^+^, an adsorbed intermediate H^*^, and gaseous H_2_ molecule as the final product. Figure 4b showed the free energy diagram towards acidic HER of Os SACs with various coordination environments, of which structure images for hydrogen transfer process were shown in Supplementary Fig. 32a–f. The adsorption free energies of adsorbed intermediates were displayed in Supplementary Tables 4, 5. From the kinetic viewpoint, the highest reaction barrier among hydrogen transfer process is known as the kinetics of rate-determining step to describe the HER catalytic activity. Δ*G*_H*_ was applied to correlate with energy barrier of atomic hydrogen capture/desorption process (Fig. 4c). When Δ*G*_H*_ > 0, H^+^ can be more easily captured from the electrolyte and combined with adsorbed H to form gaseous H_2_ with higher Δ*G*_H*_. Therefore, the acidic HER kinetics of Os SACs was controlled by oxidation state which adjusts Δ*G*_H*_. We also applied microkinetics model and converted results in free energy diagram into polarization curve simulation (Supplementary Fig. 33). Supplementary Fig. 34 and Fig. 4d directly displayed an obvious volcanic diagram between the energy barrier (or theoretical overpotential in 10 mA/cm^2^) and oxidation state of Os SACs. Therefore, Os-N_3_S_1_ with a moderate oxidation state demonstrates the lowest reaction barrier of rate-determining step and accordingly lowest overpotential among the five Os SACs in acidic HER. Besides, we also considered the possibility of Volmer-Tafel mechanism. According to the free energy diagram in Supplementary Fig. 35a, the free energy change of potential-determining step through Volmer-Tafer mechanism still showed volcano relationship with oxidation state among diverse Os SACs in Supplementary Fig. 35b, where Os-N_3_S_1_ displayed highest HER activity due to the moderate oxidation state.

**Fig. 5:**
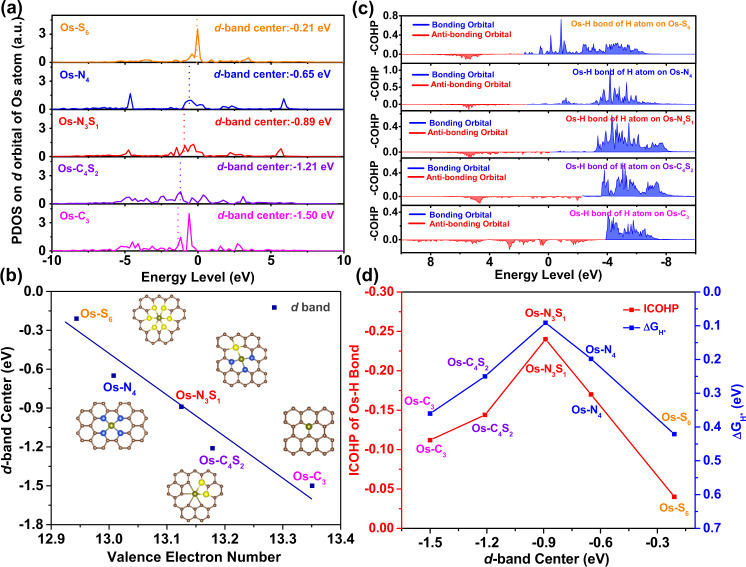
Mechanistic investigations. **a** Projected density of state for *d* orbital of Os single atom with various coordination environments. **b** *d*-band center of Os single atom as a function of valence electron number of Os single atom. **c** Crystal orbital Hamilton population (COHP) of Os-H bond for H^*^ on Os SACs. **d** Δ*G*_H*_ and integral COHP (ICOHP) of Os-H bond for H* on Os SACs as a function of *d*-band center of Os single atom.

To explore the intrinsic regulation mechanism of oxidation state for ΔG_H*_ from a point of electron structures, we turned to focus on *d*-orbital which is the only unsaturated orbital of Os element. The *d*-band centers of Os SACs with different coordinations were derived from their projected density of state (PDOS) for Os *d*-orbitals (Fig. 5a). The relative order of integration of PDOS in *d*-orbital under Fermi energy, representing the degree of valence electron occupation in *d*-orbital, among Os SACs with different coordination (Supplementary Table 6), was consistent with that derived from Bader charge analysis. As presented in Fig. 5b, the less occupied *d*-orbital with fewer valence electrons (higher oxidation state) resulted in a higher *d*-band center, which yielded an increasing energy level of the molecular orbital of Os-H bond from partially occupied anti-bonding states to bonding state with unsaturated occupancy (Fig. 5c). The change of hydrogen adsorption strength was determined by the anti-bonding states of Os-H bond at first. The decreasing number of valence electrons induced anti-bonding states with higher level and lower occupancy, leading to a stronger Os-H interaction. When valence electron number kept decreasing, the hydrogen adsorption strength converted to be affected by bonding state mainly near the Fermi level. Consequently, the upshift of bonding orbital resulted in a decreasing occupancy and receded H* adsorption strength. The value of integral crystal orbital Hamilton populations (ICOHP), reflecting Os-H bond strength, showed a volcanic curve relationship with *d*-band center (Fig. 5d). It was consistent with the analysis on Os-H bond strength (Δ*G*_H*_). Finally, coordination-dependent activity of isolate Os sites towards acidic HER was attributed to the adjusted energy-level of *d*-orbital, which was induced by oxidation state (or valence electron number) of Os single atoms. Thus, the oxidation state of isolated sites directly affected atomic hydrogen adsorption and molecular hydrogen formation.

## Discussion

In summary, this work discloses that the HER performance of Os SACs with different coordination environments was closely correlated to the oxidation state of metal atoms from both experimental results and DFT calculations. Firstly, we prepared various Os SACs with different oxidation states by regulating coordination environments, including Os-N_3_S_1_, Os-N_4_, Os-C_4_S_2_, Os-S_6_, and Os-C_3_. Notably, experimental results confirmed that a volcanic relation was obtained between the oxidation state of different Os SACs and HER catalytic activity. Moreover, Os-N_3_S_1_ with a moderate experimental oxidation state of +1.3 showed the most accelerated reaction kinetics and promising catalytic activity for acidic HER. Further intrinsic analyses revealed that the valence electron number of isolated Os atom, macroscopically reflected by oxidation state, was responsible for adjusting the energy-level of *d*-orbital to affect the atomic hydrogen adsorption and molecular hydrogen formation in acidic HER. Finally, this study not only offers a series of Os SACs with various coordination structures for hydrogen production, but also provides a fundamental understanding on the role of oxidation state in coordination-dependent activity. It may be broadened to other SACs and offers valuable guidance for rational design of SACs through engineering oxidation states.

## Methods

### Synthesis of different carbon materials

1 g activated carbon and 5 g urea were mixed in 80 mL deionized water. Then, the mixture was heated to 180 °C for 12 h in a Teflon-lined stainless steel autoclave (100 mL). After that, the mixture was dried under 50 °C and fully ground. Besides, the mixture was calcined in a tube furnace at 800 °C for 2 h under N_2_ atmosphere. Finally, N doped carbon material was successfully synthesized (defined as CN). 1 g activated carbon and 3 g 2, 2-bithiophene were fully ground. Then, the mixture was calcined in a tube furnace at 800 °C for 2 h under N_2_ atmosphere. Finally, S-doped carbon material was successfully synthesized (defined as CS). Activated carbon (1 g) and urea (5 g) were mixed in deionized water (80 mL). Then, the mixture was heated to 180 °C for 12 h in a Teflon-lined stainless steel autoclave (100 mL). After that, the mixture was dried at 50 °C and fully ground with 3 g of 2, 2-bithiophene. Next, the mixture was calcined in a tube furnace at 800 °C for 2 h under N_2_ atmosphere. Finally, N, S co-doped carbon material was successfully synthesized (defined as CNS).

### Synthesis of atomically dispersed Osmium catalysts

C/CS/CN/CNS (30 mg) and Pluronic F127 (30 mg) were fully mixed in deionized water (30 mL). Then, 0.015 mmol OsCl_3_ was dissolved in 5 mL deionized water. After that, the OsCl_3_ solution was doped into above suspension. Next, the mixture was fully mixed for 6 h and was centrifuged at 1300 g (acceleration of gravity) for 7 min. Then, the obtained products were washed with deionized water three times and were dried in a vacuum drying oven. Moreover, these samples were calcined at 400 °C for 2 h and the prepared catalysts should be washed with hydrochloric acid for 12 h. Finally, Os/C, Os/CN, Os/CS, and Os/CNS were obtained, respectively.

To obtain Os/CS-2 sample, 30 mg CS and 30 mg F127 were fully stirred in 30 mL water. Then, 0.015 mmol OsCl_3_ was dissolved in 5 mL deionized water. After that, the OsCl_3_ solution was doped into the above mentioned mixture. Next, the mixture was fully mixed for 6 h. The obtained products were washed with deionized water for three times and were dried in a vacuum drying oven. Moreover, a porcelain boat containing sulfur powder (upstream) and a porcelain boat including sample were calcined at 400 °C under N_2_ atmosphere for 2 h, simultaneously. Then, the prepared catalysts were dealt with hydrochloric acid for 12 h. Finally, atomically dispersed Os/CS-2 was synthesized.

### Synthesis of Osmium nanoparticles supported on CNS

30 mg CNS was uniformly dispersed in water (20 mL) under magnetic stirring. Then, 0.06 mmol OsCl_3_ was dissolved in 5 mL deionized water. After that, the OsCl_3_ solution was doped into the beaker which contained CNS. Next, the mixture was fully mixed for 6 h and was centrifuged at 1300 g for 7 min. Then, the obtained product was dried in a vacuum drying oven. Finally, the obtained products were calcined in tube furnace under N_2_ atmosphere at 600 °C for 2 h.

### Characterizations

The morphology and structure of nanomaterials were observed on a H800 transmission electron microscope (TEM, JEM-2100F) which is coupled with energy dispersive X-ray spectroscopy (EDS) with an acceleration voltage of 200 kV. Besides, the HAADF-STEM images and EDS elemental mapping of electrocatalyst were carried out in a JEOL ARM-200 microscope at 200 kV, equipped with a probe spherical aberration corrector. The X-ray diffraction (XRD) was recorded on X’Pert PRO MPD (D8) instrument to detect the phase composition of samples. Besides, the surface characteristics of materials were tested by X-ray photoelectron spectroscopy (XPS) carried out on ESCALAB 250 XI system with an Mg-Ka source. The X-ray absorption find structure spectra (Os *L*-edge) were collected at 1W1B station in Beijing Synchrotron Radiation Facility (BSRF). The storage rings of BSRF were operated at 2.5 GeV with a maximum current of 250 mA. Using Si (111) double-crystal monochromator, the data collection was carried out in transmission mode using ionization chamber. All spectra were collected in ambient conditions. The soft XANES spectra (C *K*-edge and N *K*-edge) were measured at beamline BL12B of National Synchrotron Radiation Laboratory (NSRL).

### Electrochemical measurements

The electrochemical measurements were carried out in a three-electrode system controlled by CHI 760E (CHEN HUA, China) electrochemistry workstation. The obtained electrocatalyst (5 mg) were mixed with water (0.2 mL), ethanol (0.8 mL) and Nafion (15 µL). A homogeneous ink was gained after sonication for 30 min. Besides, a graphite rod was served as counter electrode. Saturated calomel electrode was used as the reference electrode. Then, 15 µL of catalyst ink was deposited on glassy-carbon electrode to obtain working electrode after the solvent was dried naturally. In this study, linear sweep voltammetry (LSV) curves for HER were carried out in 0.5 M H_2_SO_4_ media with a scan rate of 5 mV s^−1^. The electrochemical impedance spectroscopy (EIS) tests were recorded with frequency from 0.1 to 100000 Hz.

### Calculation of turnover frequency (TOF)

TOF per metal site of the catalysts was shown according to the following equation:

1 $${{{\rm{TOF}}}}=\left|{{{\rm{J}}}}\right|/(2{{{\rm{nF}}}})$$

*J* is the current density (A cm^−2^) from the LSV measurement, n is the number of active sites per geometric area (mol cm^−2^), F is the Faraday constant (96485.33 C mol^−1^). The factor 1/2 derives from the fact that two electrons are required for one hydrogen molecule. The Os content of catalysts is determined by the ICP-OES.

### XAFS analysis

The acquired EXAFS data were processed according to the standard procedures using the ATHENA module implemented in the IFEFFIT software packages. The k^3^-weighted EXAFS spectra were obtained by subtracting the post-edge background from the overall absorption and then normalizing with respect to the edge-jump step. Subsequently, k^3^-weighted χ(k) data of Os *L*-edge were Fourier transformed to real (R) space using a hanning windows (d_k_ = 1.0 Å^−1^) to separate the EXAFS contributions from different coordination shells. To obtain the quantitative structural parameters around central atoms, least-squares curve parameter fitting was performed using the ARTEMIS module of IFEFFIT software packages.

### DFT computational details

All DFT calculations with consideration of spin-polarization were conducted by using first-principle calculations, as implemented in the Vienna ab initio Simulation Package (VASP) code^47,48^. Meanwhile, we used the Perdew-Burke-Ernzerhof (PBE) functional within the generalized gradient approximation (GGA) to describe the exchange correlation energy^49^. We used the projector augmented wave (PAW) pseudo-potentials to depict the interaction between valence electron and ionic cores^50^. The Kohn-Sham equations was expanded in a plane wave basis set with cutoff energy of 500 eV after a series of test. The electronic orbital occupancies were determined in light of gaussian smearing of 0.05 eV during the geometry optimization and for the total energy computations, whilst a tetrahedron method with Blöchl corrections was applied in the accurate electronic structure calculations. The convergence threshold for the iteration in self-consistent-field (SCF) was set to be 10^−6^ eV, and that for geometry optimizations by BFGS algorithm was set to be 0.01 eV/Å on maximum force component. All of the Os SACs supported on the graphene models are constructed based on the graphene basal plane model with the supercell of 5 × 5 (12.30 Å × 12.78 Å), with a large vacuum slab of 15 Å inserted in z-direction for surface isolation to prevent interaction between two neighboring surfaces. The Monkhorst-Pack meshes of 3 × 3 × 1 k-point samplings in the Brillouin zones were used for these models. Denser k-points (12 × 12 × 1) were used for the electronic structure calculations. Considering that the solvent may influence the adsorption free energy, we employed VASPsol to include the implicit solvation effect in the calculations^51^. The vibrational frequencies of free molecules and adsorbates, which are needed to determine zero-point energies and vibrational entropies, were calculated by using the phonon modules in VASP 5.3 code. The Reaction free energy calculation of HER on Os single atom catalysts supported on graphene were calculated according to electrochemical framework of calculation hydrogen electrode developed by Nørskov, which can be referred to our previous works^52^. The climbing-image nudged elastic band (CI-NEB) method was used to determine transition states and reaction paths. Through vibrational frequency analyses, all transition states have been verified by ensuring that they have only one non-negligible imaginary vibrational frequency.

### Microkinetics model and polarization curve simulation

The Volmer-Heyrovsky pathway are listed by the following equations:

2 $$\ast+{H}^{+}+{e}^{-}\mathop{\longleftrightarrow }\limits^{{k}_{1},{k}_{-1}}{H}^{\ast }$$

3 $${H}^{\ast }+{H}^{+}+{e}^{-}\mathop{\longleftrightarrow }\limits^{{k}_{2},{k}_{-2}}{H}_{2}+\ast $$

where the adsorption site of hydrogen is denoted by Asterisks. Note that Eqs. (2) and (3) are both electrochemical step. Based on the above reduction step, we can gain the rate equations along with the site conservation condition:

4 $$\frac{{\theta }_{H*}}{\partial t}={k}_{1}{\theta }_{*}-{{k}_{1}\theta }_{H*}-{k}_{2}{\theta }_{H*}+{k}_{-2}{\theta x}_{H2}$$

here, $\theta$ is the coverage of the species. The coverage of free sites may be approximated by an effective Langmuir isotherm: ${\theta }_{\ast }+{\theta }_{H\ast }=1$. *k*_*i*_ is the rate constants. The rate equation can be solved numerically at steady state, and further infer the turn over frequency (TOF). For electrochemical step, the equilibrium constant (*K*_*i*_), which is associated with the reaction potential (U vs. RHE), can be expressed by

5 $${K}_{i}={{\exp }}\left(-\frac{e(U-{U}_{i})}{{k}_{B}T}\right)$$

where ${U}_{i}$ is the reversible potential of step i deduced by ${U}_{i}=-\varDelta {G}_{i}/e$. And the *k*_*i*_ is written as:

6 $${k}_{i}={A}_{i}{{\exp }}\left(-\frac{{E}_{a,i}}{{k}_{B}T}\right){{\exp }}\left(-\frac{e{\beta }_{i}(U-{U}_{i})}{{k}_{B}T}\right)$$

where ${A}_{i}$ is an effective pre-exponential factor taken as 10^9^ s^−1^, and the ${\beta }_{i}$ is the symmetric factor taken to be 0.5. *E*_*a,i*_ is energy barrier of elementary step. Moreover, the rate constants for all the reverse reaction (${k}_{-i}$) can be expressed by:

7 $${k}_{-i}=\frac{{k}_{i}}{{K}_{i}}$$

Finally, the current density (j) can be gained by:

8 $${{{\rm{j}}}}=-2{{{\rm{e}}}}{{{\rm{\rho }}}}{{TOF}}_{H2}$$

where e is the elementary charge and ρ is the surface density of active sites.

## Supplementary information

Supplementary Information

## Data Availability

All data is available in the main text or the supplementary information. Source data are provided with this paper.

## Source data

Source Data

## References

[1] Zhang Z (2020). Electrochemical deposition as a universal route for fabricating single-atom catalysts. Nat. Commun..

[2] Zhao D (2020). Atomic site electrocatalysts for water splitting, oxygen reduction and selective oxidation. Chem. Soc. Rev..

[3] Qi H (2021). Highly selective and robust single-atom catalyst Ru_1_/NC for reductive amination of aldehydes/ketones. Nat. Commun..

[4] Qiao M (2020). Hierarchically ordered porous carbon with atomically dispersed FeN_4_ for ultraefficient oxygen reduction reaction in proton-exchange membrane fuel cells. Angew. Chem. Int. Ed..

[5] He X (2019). A versatile route to fabricate single atom catalysts with high chemoselectivity and regioselectivity in hydrogenation. Nat. Commun..

[6] Li S (2020). Impact of the coordination environment on atomically dispersed Pt catalysts for oxygen reduction reaction. ACS Catal..

[7] Yang H (2021). Manganese vacancy-confined single-atom Ag in cryptomelane nanorods for efficient wacker oxidation of styrene derivatives. Chem. Sci..

[8] Gao R (2021). Pt/Fe_2_O_3_ with Pt-Fe pair sites as a catalyst for oxygen reduction with ultralow Pt loading. Nat. Energy.

[9] Xia C (2021). General synthesis of single-atom catalysts with high metal loading using graphene quantum dots. Nat. Chem..

[10] Yang H (2019). A universal ligand mediated method for large scale synthesis of transition metal single atom catalysts. Nat. Commun..

[11] Cui X (2018). Bridging homogeneous and heterogeneous catalysis by heterogeneous single-metal-site catalysts. Nat. Catal..

[12] Cao D (2021). Construction of dual-site atomically dispersed electrocatalysts with Ru-C_5_ single atoms and Ru-O_4_ nanoclusters for accelerated alkali hydrogen evolution. Small.

[13] Lang R (2020). Single-atom catalysts based on the metal-oxide interaction. Chem. Rev..

[14] Su H (2020). Single atoms of iron on MoS_2_ nanosheets for N_2_ electroreduction into ammonia. Angew. Chem. Int. Ed..

[15] Li J-C (2019). Secondary-atom-assisted synthesis of single iron atoms anchored on N-doped carbon nanowires for oxygen reduction reaction. ACS Catal..

[16] Fei H (2018). General synthesis and definitive structural identification of MN_4_C_4_ single-atom catalysts with tunable electrocatalytic activities. Nat. Catal..

[17] Shi Z (2021). Confined Ir single sites with triggered lattice oxygen redox: toward boosted and sustained water oxidation catalysis. Joule.

[18] Jiang K (2021). Rational strain engineering of single-atom ruthenium on nanoporous MoS_2_ for highly efficient hydrogen evolution. Nat. Commun..

[19] Zhou P (2021). Partially reduced Pd single atoms on CdS nanorods enable photocatalytic reforming of ethanol into high value-added multicarbon compound. Chemistry.

[20] Liu J (2020). Rare earth single-atom catalysts for nitrogen and carbon dioxide reduction. ACS Nano..

[21] Wang L (2017). Supported rhodium catalysts for ammonia-borane hydrolysis: dependence of the catalytic activity on the highest occupied state of the single rhodium atoms. Angew. Chem. Int. Ed..

[22] Duan Z (2020). Surface charge and electrostatic spin crossover effects in CoN_4_ electrocatalysts. ACS Catal..

[23] Wang Y (2021). Boosting nitrogen reduction to ammonia on FeN_4_ sites by atomic spin regulation. Adv. Sci..

[24] Shi Y (2021). Electronic Metal-support interaction modulates single-atom platinum catalysis for hydrogen evolution reaction. Nat. Commun..

[25] Miao J (2021). Spin-state-dependent peroxymonosulfate activation of single-Atom M-N moieties via a radical-free pathway. ACS Catal..

[26] Sun S (2019). Boosting oxygen evolution kinetics by Mn-N-C motifs with tunable spin state for highly efficient solar-driven water splitting. Adv. Energy Mater..

[27] Jeong H (2020). Controlling the oxidation state of Pt single atoms for maximizing catalytic activity. Angew. Chem. Int. Ed..

[28] Ren Y (2019). Unraveling the coordination structure-performance relationship in Pt_1_/Fe_2_O_3_ single-atom catalyst. Nat. Commun..

[29] Pan Y (2019). Regulating the coordination structure of single-atom Fe-N_x_C_y_ catalytic sites for benzene oxidation. Nat. Commun..

[30] Bao H (2021). Isolated copper single sites for high-performance electroreduction of carbon monoxide to multicarbon products. Nat. Commun..

[31] Liu H (2022). Single palladium site in ordered porous heteroatom-doped carbon for high-performance alkaline hydrogen oxidation. Appl. Catal. B Environ..

[32] Chen Y (2020). Highly productive electrosynthesis of ammonia by admolecule-targeting single Ag sites. ACS Nano..

[33] Han L (2021). Modulating single-atom palladium sites with copper for enhanced ambient ammonia electrosynthesis. Angew. Chem. Int. Ed..

[34] Xu J (2021). Atomic Fe-Zn dual-metal sites for high-efficiency pH-universal oxygen reduction catalysis. Nano. Res..

[35] Cao L (2019). Identification of single-atom active sites in carbon-based cobalt catalysts during electrocatalytic hydrogen evolution. Nat. Catal..

[36] Chatenet M (2020). Good practice guide for papers on fuel cells and electrolysis cells for the Journal of Power Sources. J. Power Sources.

[37] Hansen JN (2021). Is there anything better than Pt for HER?. ACS Energy Lett..

[38] McCrory CCL (2013). Benchmarking heterogeneous electrocatalysts for the oxygen evolution reaction. J. Am. Chem. Soc..

[39] Liu X (2018). Self-powered H_2_ production with bifunctional hydrazine as sole consumable. Nat. Commun..

[40] Zhang L (2019). Atomic layer deposited Pt-Ru dual-metal dimers and identifying their active sites for hydrogen evolution reaction. Nat. Commun..

[41] Liu Y (2020). A general route to prepare low-ruthenium-content bimetallic electrocatalysts for pH-universal hydrogen evolution reaction by using carbon quantum dots. Angew. Chem. Int. Ed..

[42] Zhu J (2020). Recent advances in electrocatalytic hydrogen evolution using nanoparticles. Chem. Rev..

[43] Shang Y (2021). Single-atom catalysis in advanced oxidation processes for environmental remediation. Chem. Soc. Rev..

[44] Li X (2020). Microenvironment modulation of single-atom catalysts and their roles in electrochemical energy conversion. Sci. Adv..

[45] Greeley J (2006). Computational high-throughput screening of electrocatalytic materials for hydrogen evolution. Nat. Mater..

[46] Han B (2020). Strong metal-support interactions between Pt single atoms and TiO_2_. Angew. Chem. Int. Ed..

[47] Kresse G (1996). Efficiency of ab-initio total energy calculations for metals and semiconductors using a plane-wave basis set. Comput. Mater. Sci..

[48] Kresse G (1996). Efficient iterative schemes for ab initio total-energy calculations using a plane-wave basis set. Phys. Rev. B.

[49] Perdew J (1996). Generalized gradient approximation made simple. Phys. Rev. Lett..

[50] Kresse G (1999). From ultrasoft pseudopotentials to the projector augmented-wave method. Phys. Rev. B.

[51] Mathew K (2014). Implicit solvation model for density-functional study of nanocrystal surfaces and reaction pathways. J. Chem. Phys..

[52] Xu H (2018). A universal principle for a rational design of single-atom electrocatalysts. Nat. Catal..

