# Supplementary material for: Volcano-type relationship between oxidation states and catalytic activity of single-atom catalysts towards hydrogen evolution
Source: Nat Commun. 2022 Oct 4;13:5843. doi: 10.1038/s41467-022-33589-y (PMC9532448; doi:10.1038/s41467-022-33589-y)
Supplement: Supplementary file 1 — Supplementary Information [file 41467_2022_33589_MOESM1_ESM.pdf]

Supplementary Information

Volcano-type relationship between oxidation states and catalytic activity of single-atom catalysts towards hydrogen evolution

Dong Cao[1], Haoxiang Xu[1], Hongliang Li[2], Chen Feng[2], Jie Zeng[2*] and Daojian Cheng[1*]

[1] State Key Laboratory of Organic-Inorganic Composites and Beijing Advanced Innovation Center for Soft Matter Science and Engineering, Beijing University of Chemical Technology, Beijing 100029, People's Republic of China

[2] Hefei National Laboratory for Physical Sciences at the Microscale, Key Laboratory of Strongly-Coupled Quantum Matter Physics of Chinese Academy of Sciences, Key Laboratory of Surface and Interface Chemistry and Energy Catalysis of Anhui Higher Education Institutes, Department of Chemical Physics, University of Science and Technology of China, Hefei, Anhui 230026, P. R. China

[*] E-mail: chengdj@mail.buct.edu.cn (D. C.); zengj@ustc.edu.cn (J. Z.)

These authors contributed equally: Dong Cao, Haoxiang Xu.

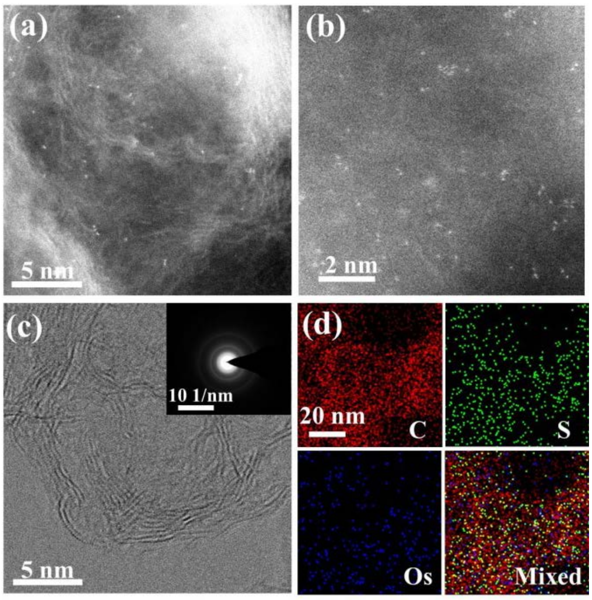

Supplementary Figure 1. **Electron microscopic characterization**. **a**, **b** AC-HAADF-STEM images of Os/CS-2 sample in different scale bars. **c** TEM image of Os/C. The inset corresponds to the SAED pattern. **d** Corresponding elemental mapping results of Os/CS-2, suggesting the uniform distribution of every element.

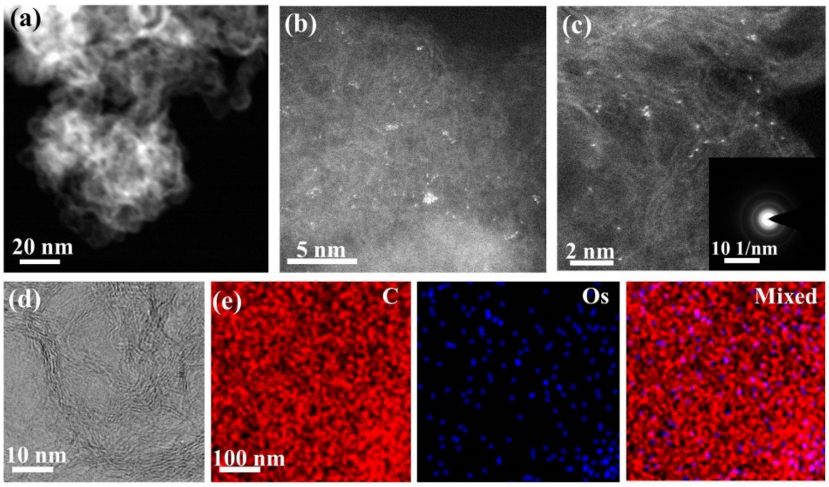

Supplementary Figure 2. **Electron microscopic characterization**. **a** STEM image of Os/C sample. **b**, **c** AC-HAADF-STEM images of Os/C. The inset corresponds to the SAED. **d** Aberration corrected TEM image of Os/C. **e** Corresponding elemental mapping results of Os/C, suggesting the uniform distribution of every element.

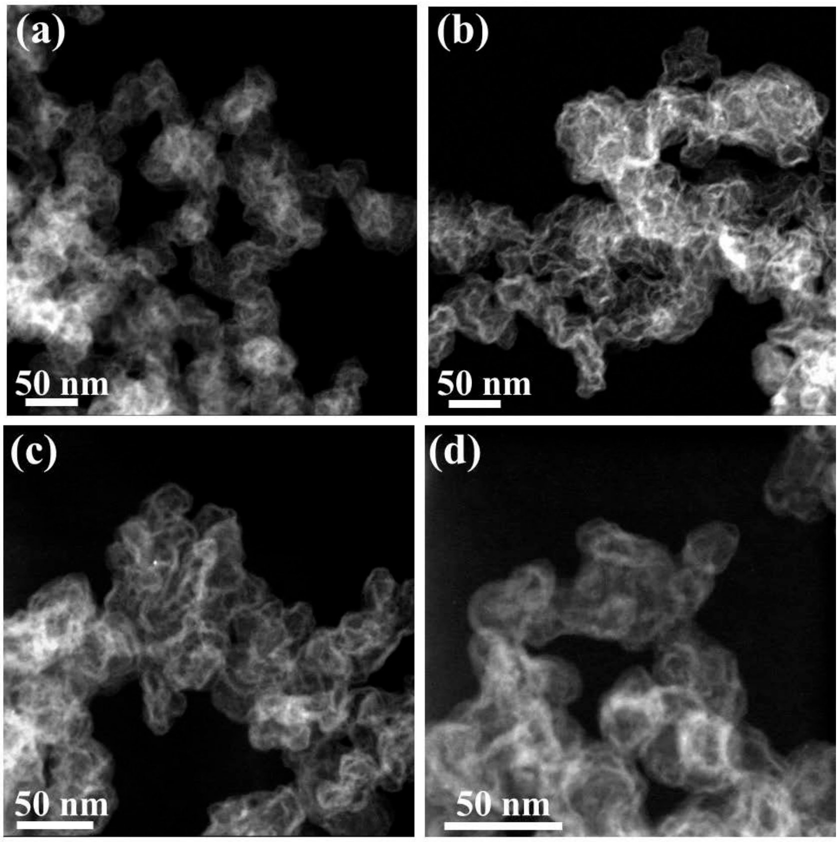

Supplementary Figure 3. **Electron microscopic characterization**. **a-d** Low resolution STEM images of Os/CNS, Os/CN, Os/CS, and Os/CS-2, respectively.

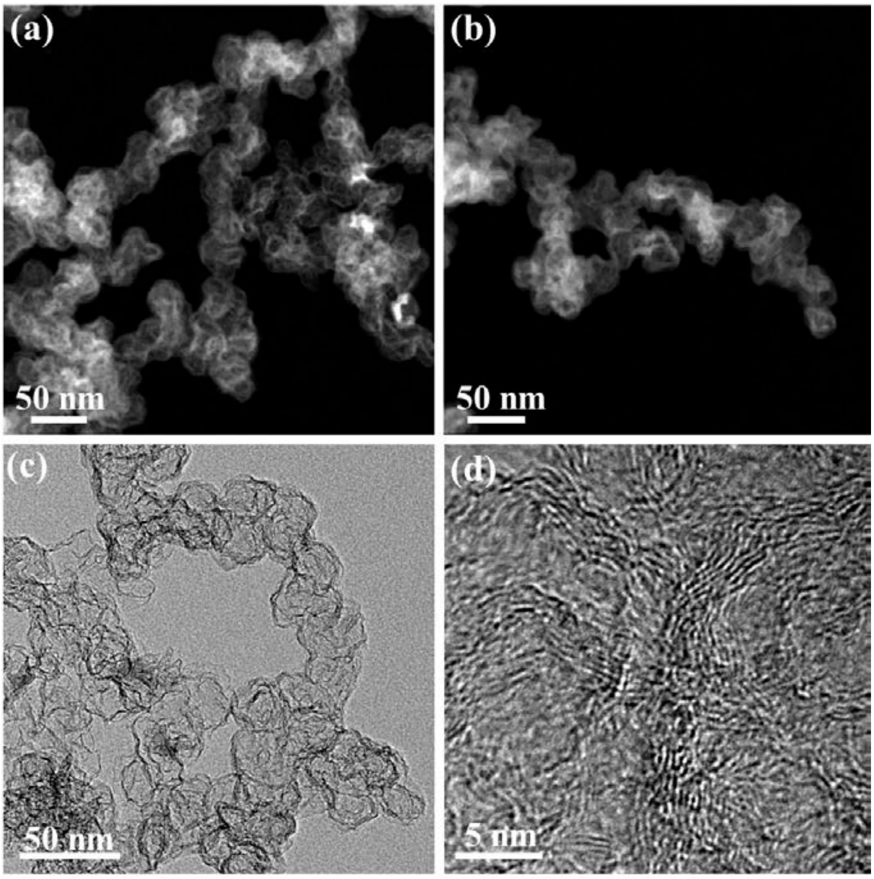

Supplementary Figure 4. **Electron microscopic characterization**. **a**, **b** STEM images of pure CNS support in different scale bars. **c** TEM image of CNS. **d** HRTEM image of CNS.

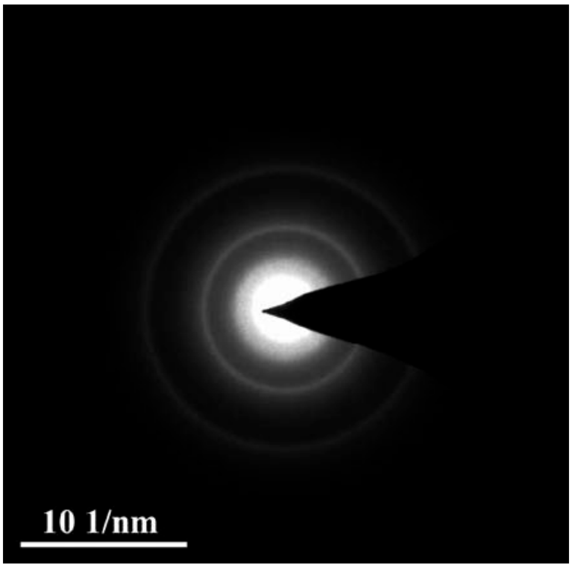

Supplementary Figure 5. **Characterization of carrier**. SAED pattern of pure CNS support.

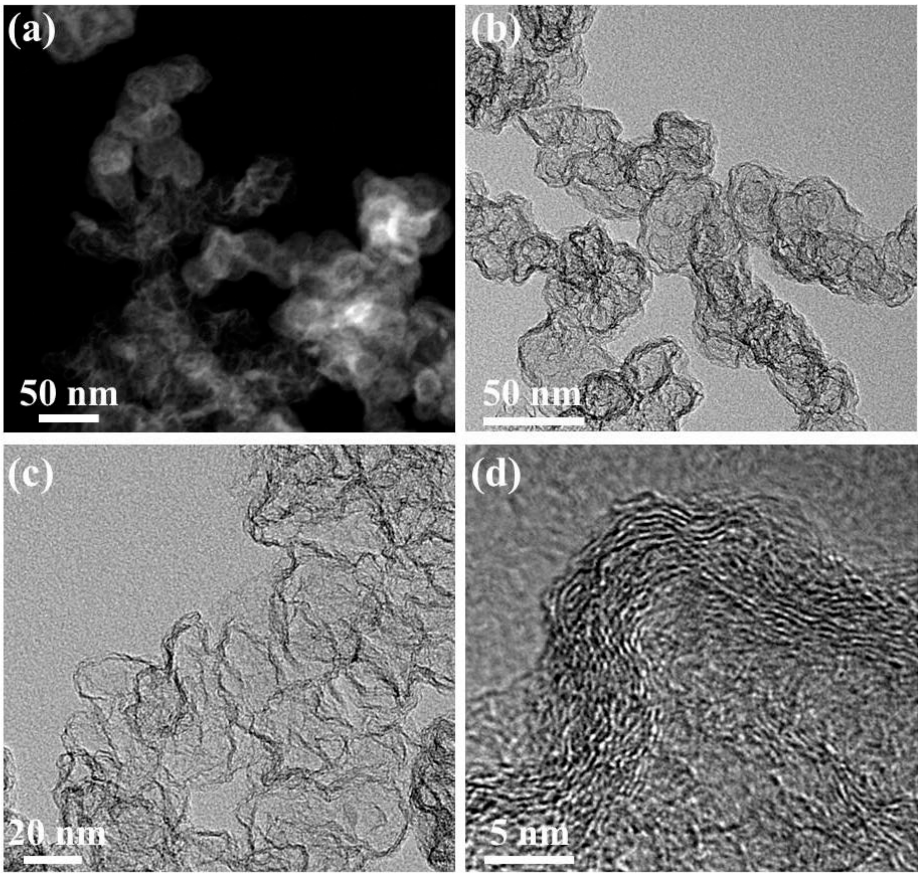

Supplementary Figure 6. **Electron microscopic characterization**. **a** STEM images of pure CN support. **b**, **c** TEM image of CN in different scale bars. **d** HRTEM image of CN.

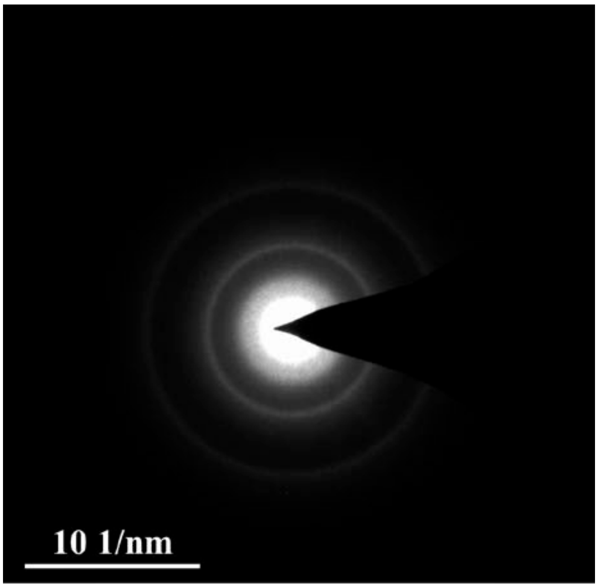

Supplementary Figure 7. **Characterization of carrier**. SAED pattern of pure CN support.

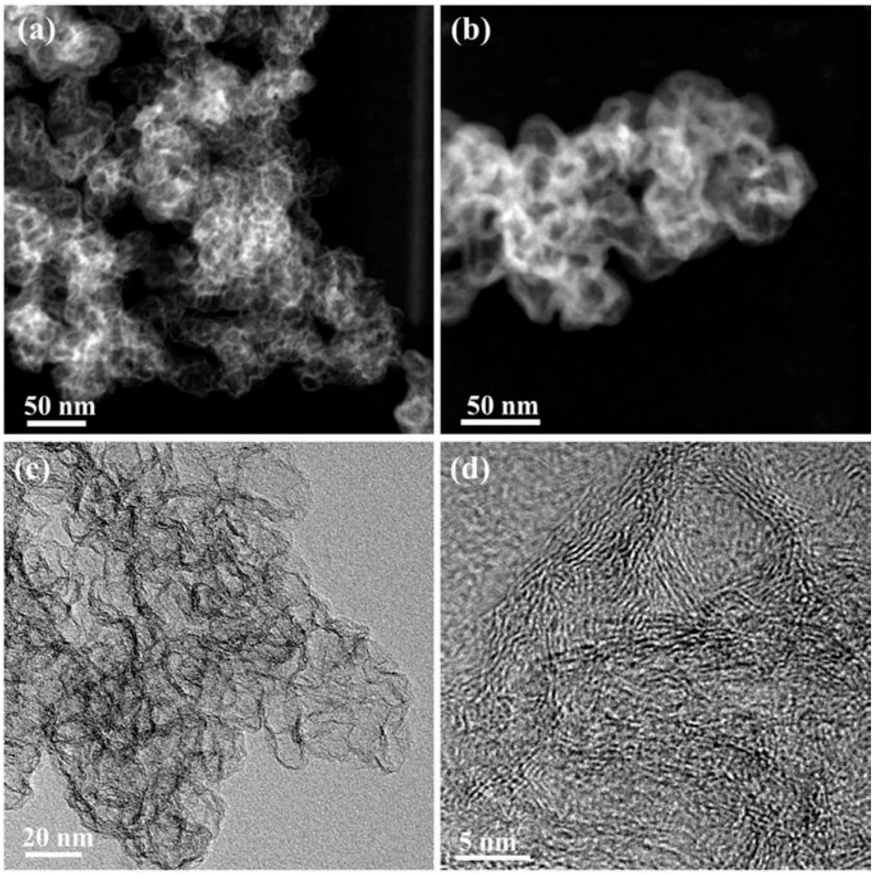

Supplementary Figure 8. **Electron microscopic characterization**. **a**, **b** STEM images of pure C support in different scale bars. **c** TEM image of C. **d** HRTEM image of C.

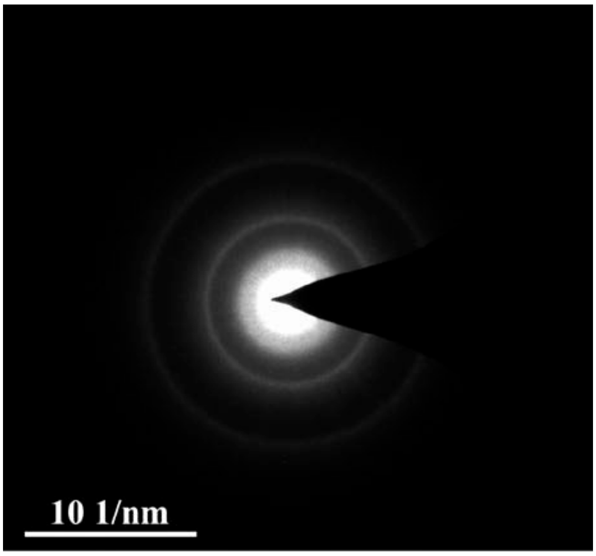

Supplementary Figure 9. **Characterization of carrier**. SAED pattern of pure C support.

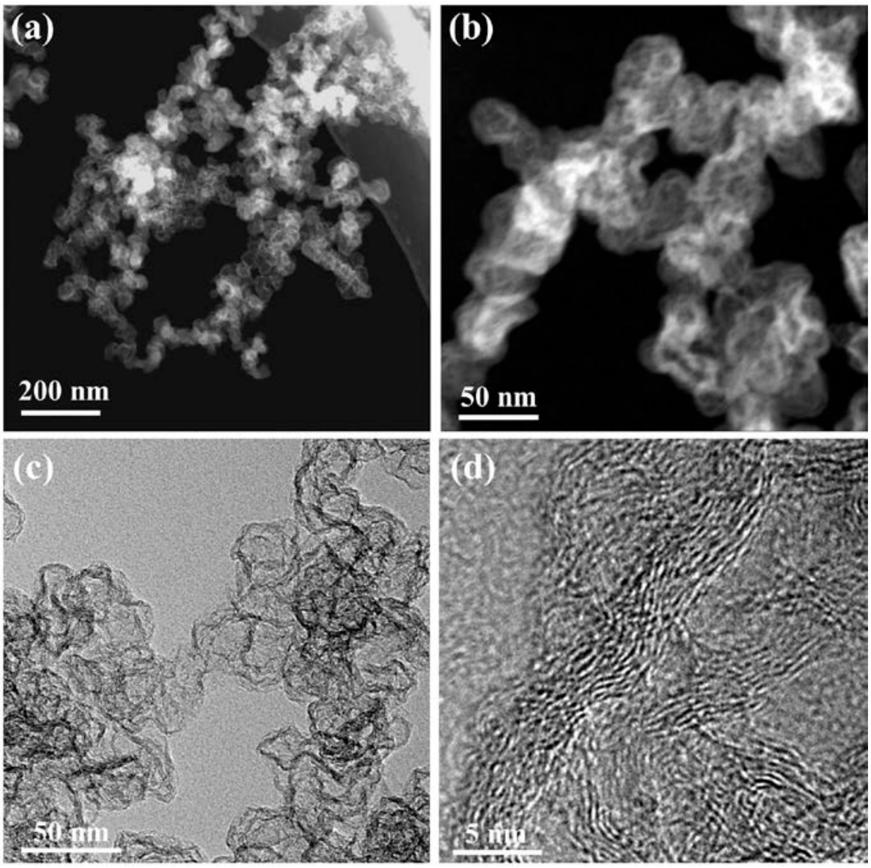

Supplementary Figure 10. **Electron microscopic characterization**. **a**, **b** STEM images of pure CS support in different scale bars. **c** TEM image of pure CS. **d** HRTEM image of pure CS.

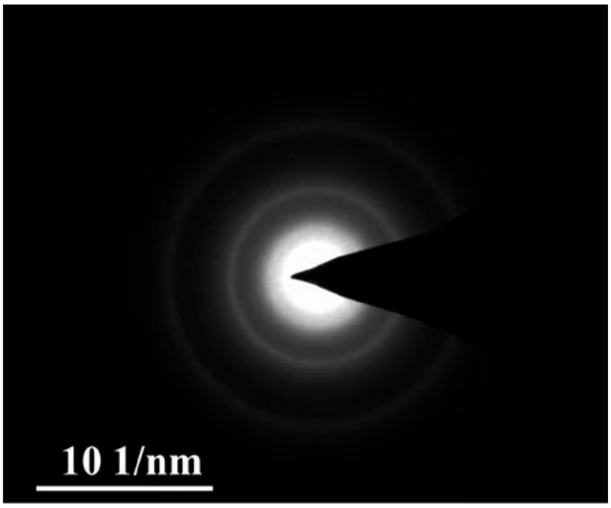

Supplementary Figure 11. **Characterization of carrier**. SAED pattern of pure CS support, which is consistent with pure carbon black.

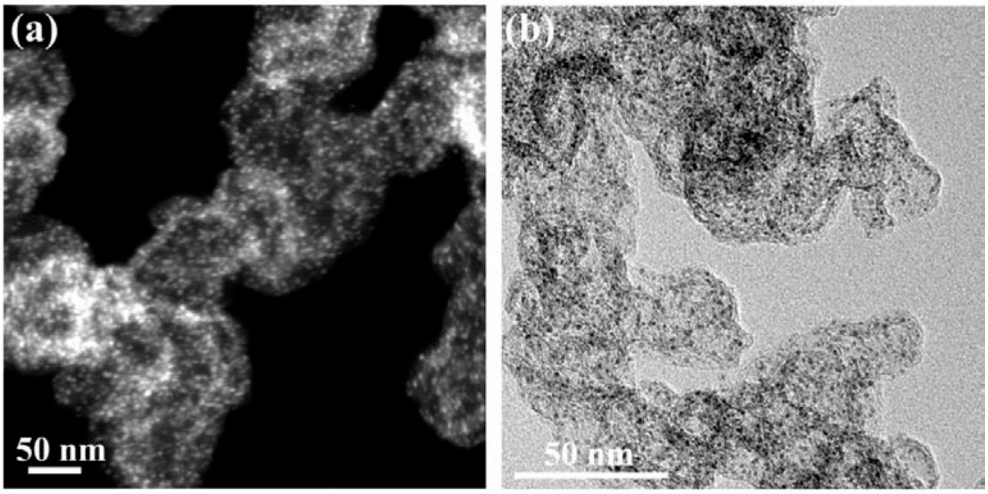

Supplementary Figure 12. **Electron microscopic characterization**. **a** STEM image of Os NPs. **b** TEM image of Os NPs.

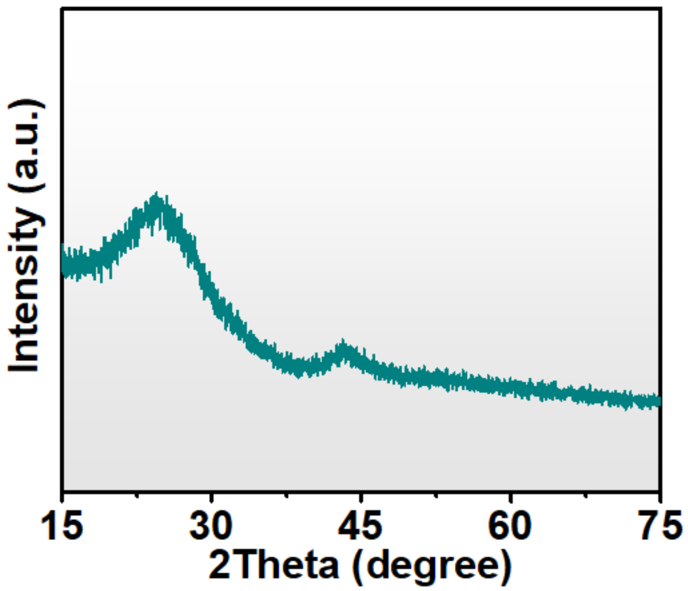

Supplementary Figure 13. **Characterization of carbon black**. XRD pattern of pure carbon black, suggesting the polycrystalline structure of carbon black.

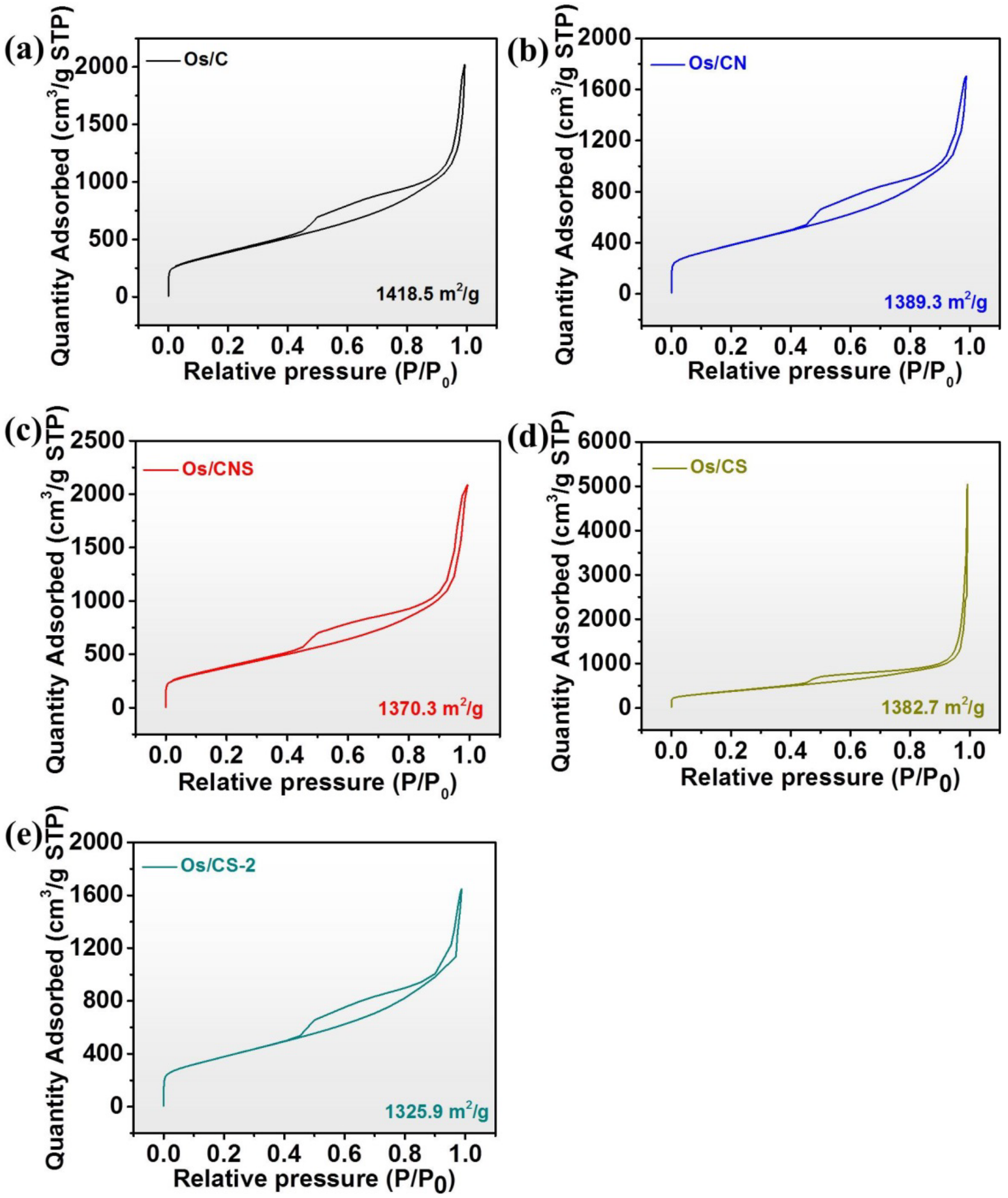

Supplementary Figure 14. **N_2 adsorption-desorption characterization**. The nitrogen adsorption-desorption isotherm of **a** Os/C, **b** Os/CN, **c** Os/CNS, **d** Os/CS, and **e** Os/CS-2 samples.

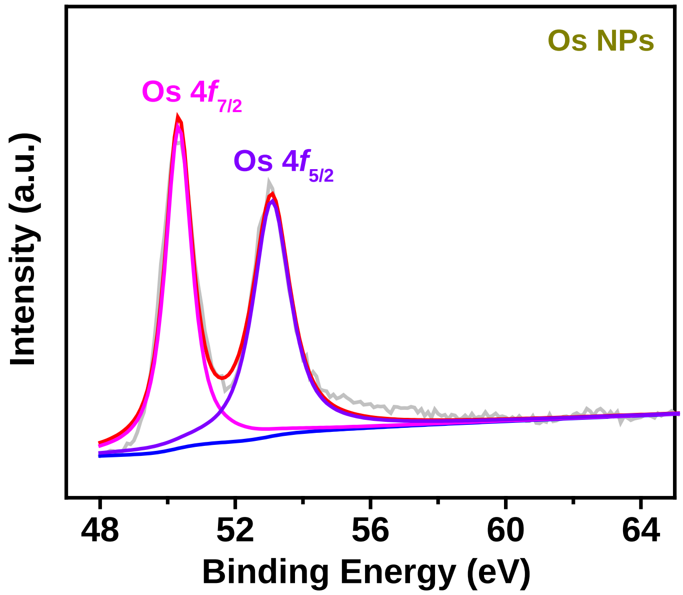

Supplementary Figure 15. **Characterization of Os nanoparticles**. XPS spectrum of Os 4*f* in Os nanoparticles (NPs).

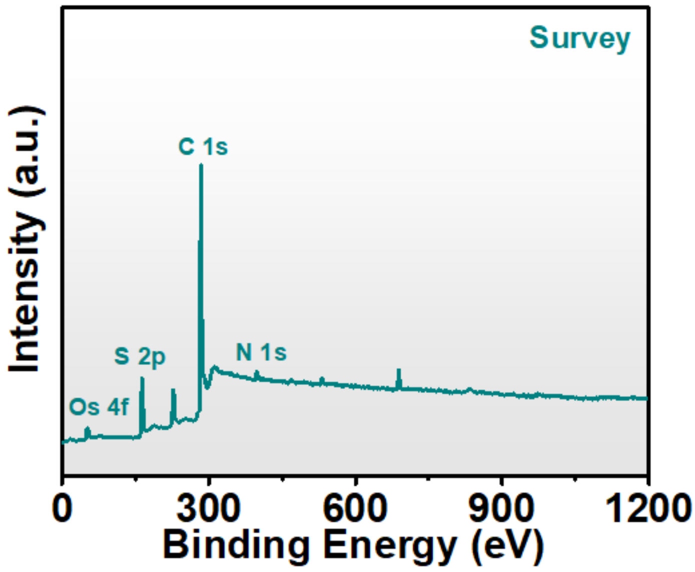

Supplementary Figure 16. **Characterization of Os/CNS**. XPS survey spectrum of Os/CNS sample, suggesting the existence of Os, C, N, and S elements.

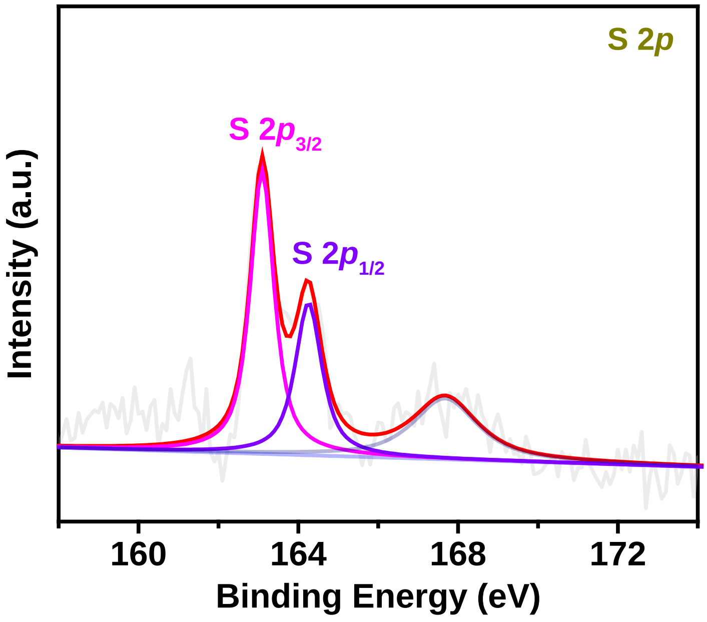

Supplementary Figure 17. **Characterization of CNS carrier**. XPS spectrum of S element for pure CNS support, revealing no Metal-S bonds can be observed.

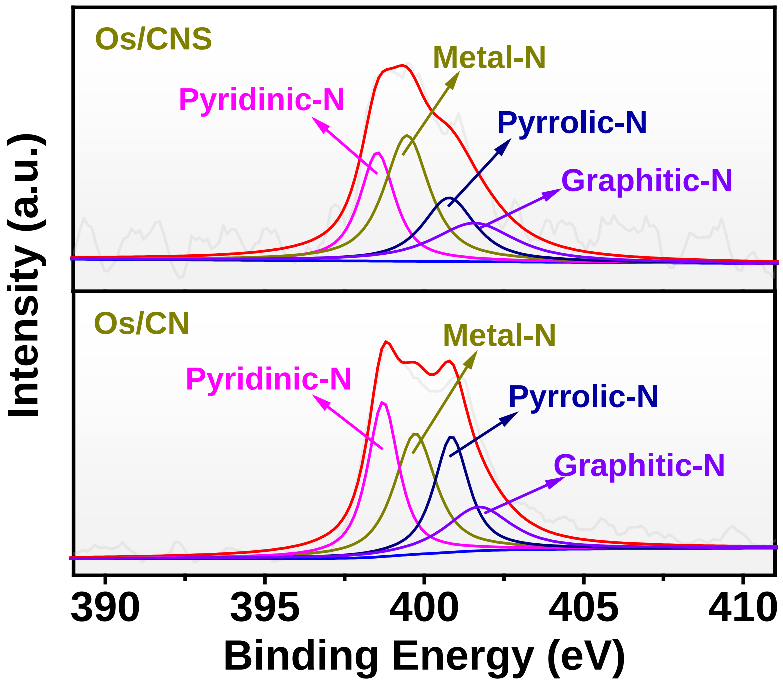

Supplementary Figure 18. **XPS for N element**. High-resolution XPS spectra of N 1*s* in different samples.

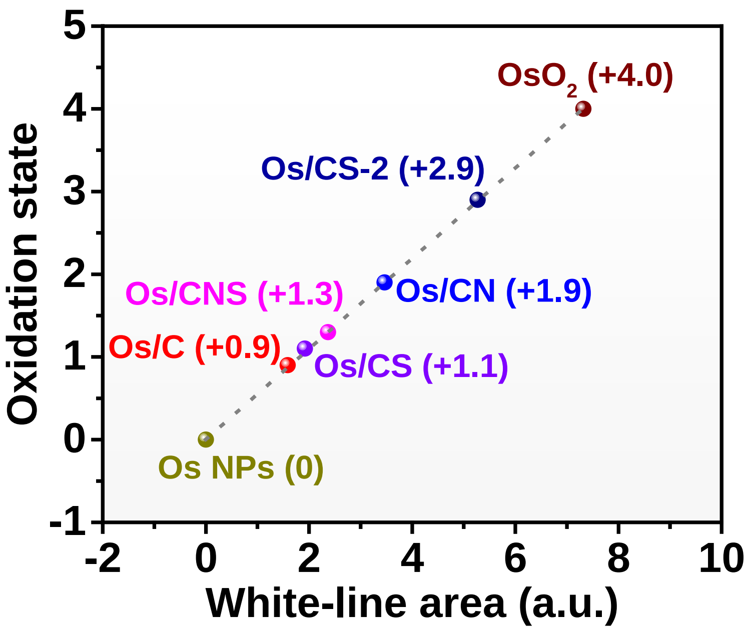

Supplementary Figure 19. **Calculation of oxidation states**. Fitted curve correlating the average oxidation state of Os for different samples at Os *L*-edge.

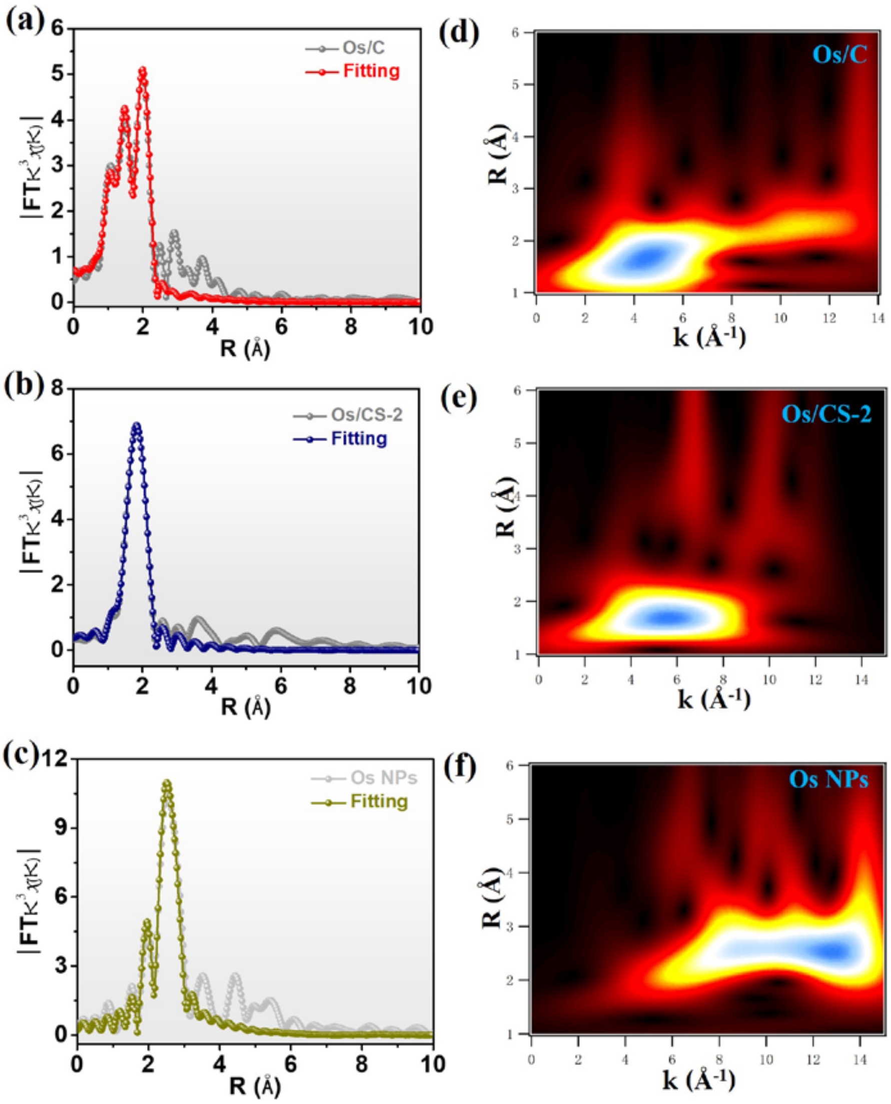

Supplementary Figure 20. **XAFS analyses**. **a-c** FT-EXAFS fitting curves of Os/C, O/CS-2, and Os nanoparticles (NPs) catalysts, respectively. **d-f** WT-EXAFS of Os/C, O/CS-2, and Os NPs, respectively.

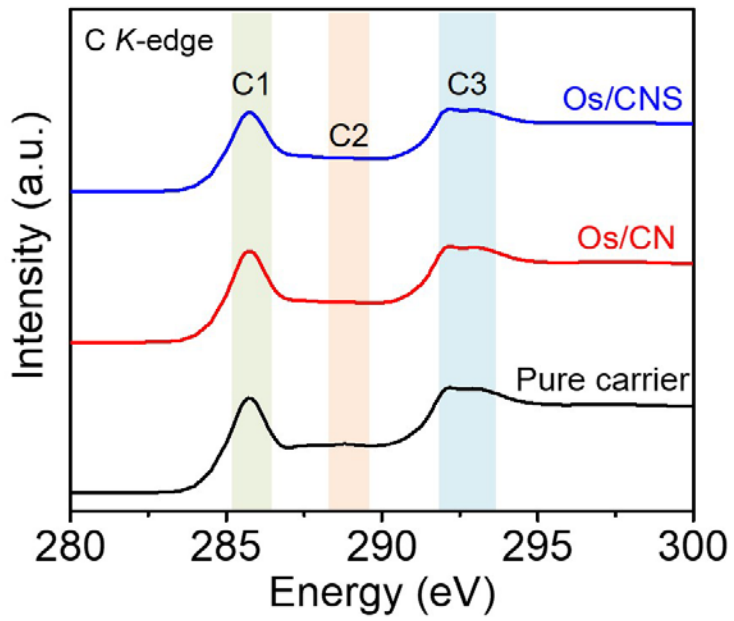

Supplementary Figure 21. X-ray absorption spectroscopy (XAS) spectra. C *K*-edge XAS spectra of different samples in this work.

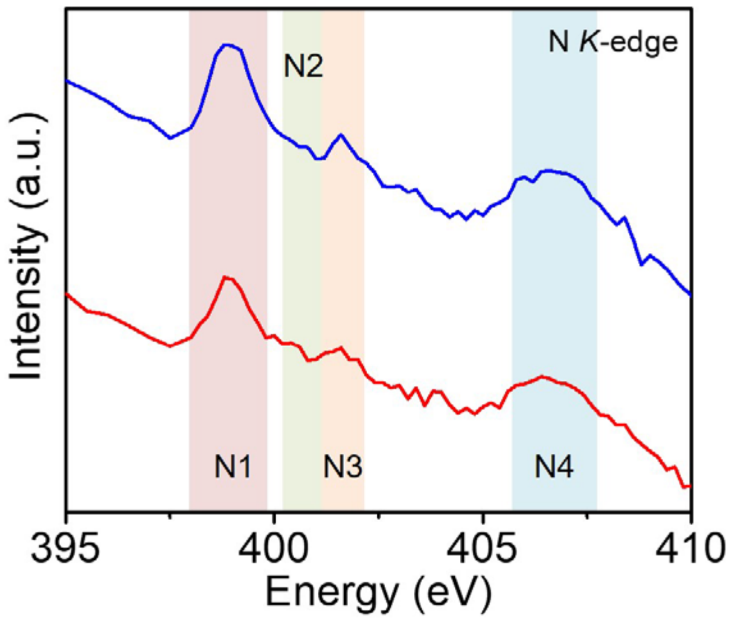

Supplementary Figure 22. X-ray absorption spectroscopy (XAS) spectra. N *K*-edge XAS spectra of different samples in this work.

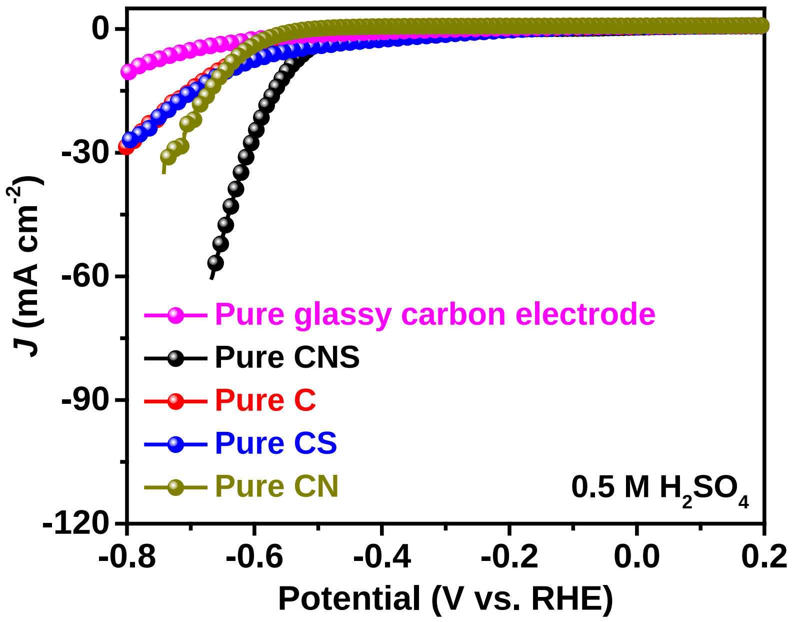

Supplementary Figure 23. **Characterizations of HER activity**. LSV curves of pure carbon materials in acidic media, revealing supports display no activity for HER.

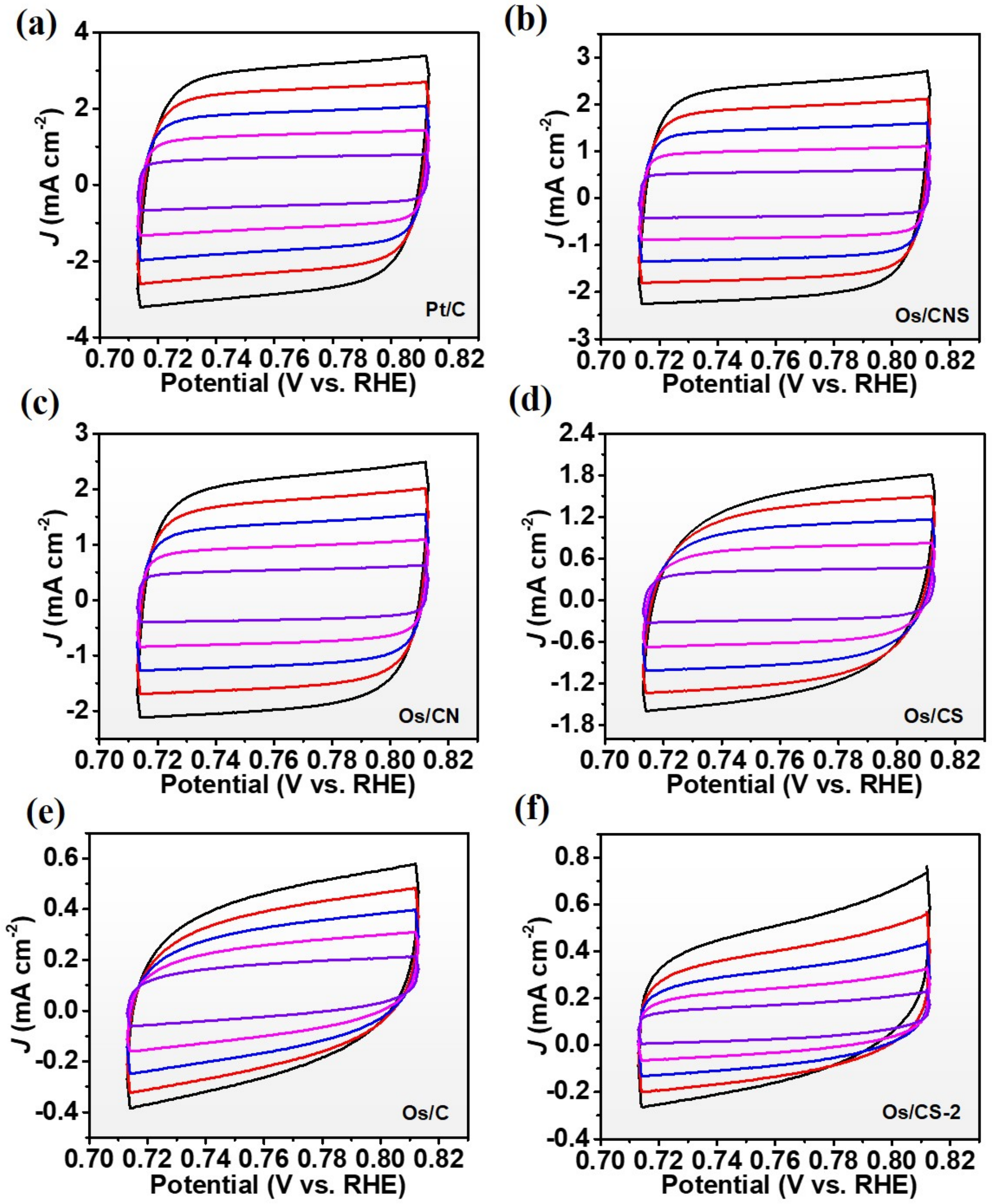

Supplementary Figure 24. **Cyclic voltammograms (CVs) of various catalysts**. **a** CVs of Pt/C. **b** CVs for Os/CNS. **c** CVs of Os/CN. **d** CVs of Os/CS. **e** CVs of Os/C. **f** CVs of Os/CS-2. The sweep rates are in the range of 20-100 mV/s.

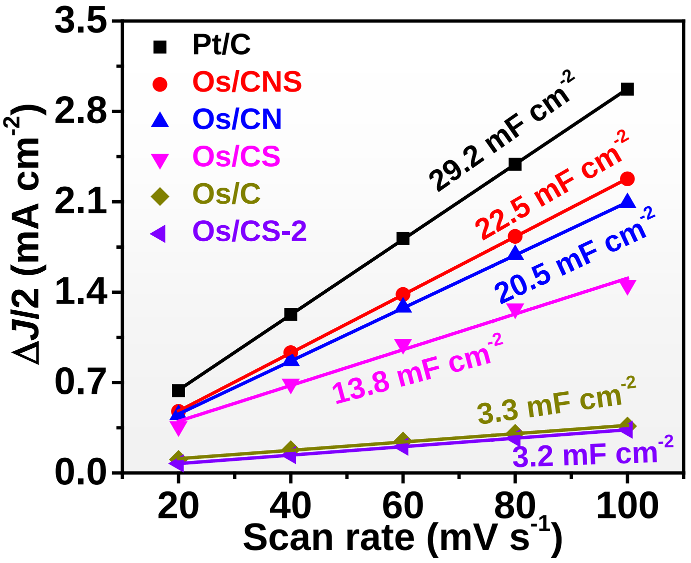

Supplementary Figure 25. **The fitting of double layer capacity (C_{dl})**. The plotted C_{dl} of electrocatalysts in this work for HER in 0.5 M H_2SO_4 solution.

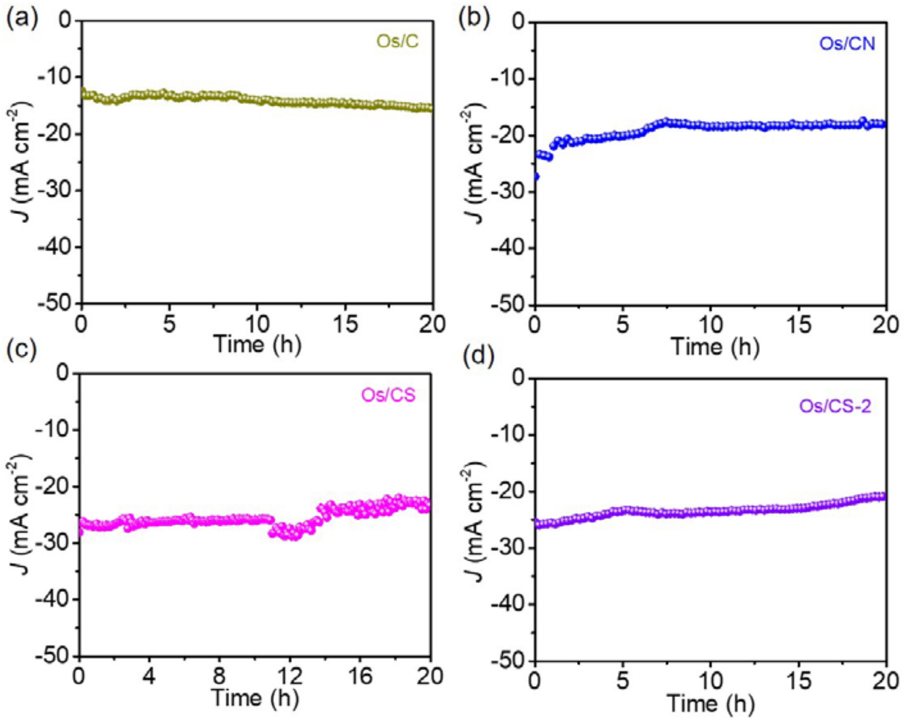

Supplementary Figure 26. **Stability tests**. **a-d** The chronoamperometric curves of Os/C, Os/CN, Os/CS, Os/CS-2 electrocatalysts in this work, respectively.

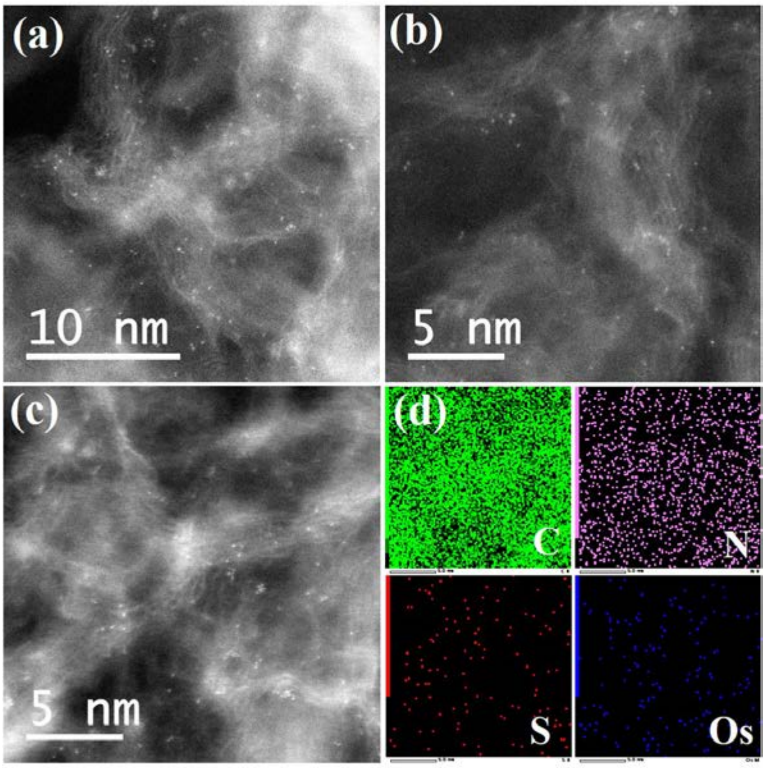

Supplementary Figure 27. Characterization of Os/CNS after HER. **a-c** Aberration corrected HAADF-STEM images of Os/CNS in different scale bars. **d** Corresponding EDS elemental mapping of Os/CNS.

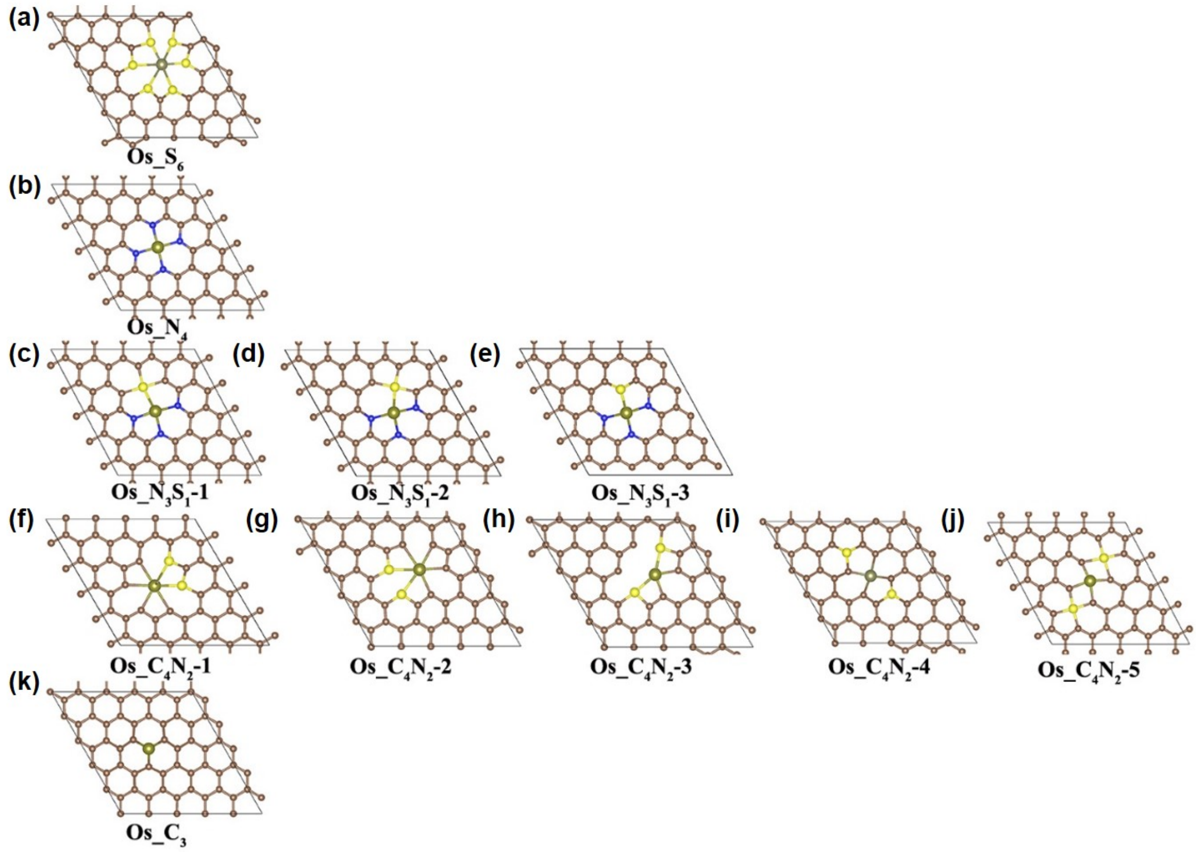

Supplementary Figure 28. **Possible configurations of different Os SACs**. **a** The coordination structure of Os-S_6. **b** The coordination structure of Os-N_4. **c-e** The three kinds of possible configurations for Os-N_3S_1. **f-j** The five kinds of possible structure of Os-C_4S_2. **k** The coordination structure of Os-C_3.

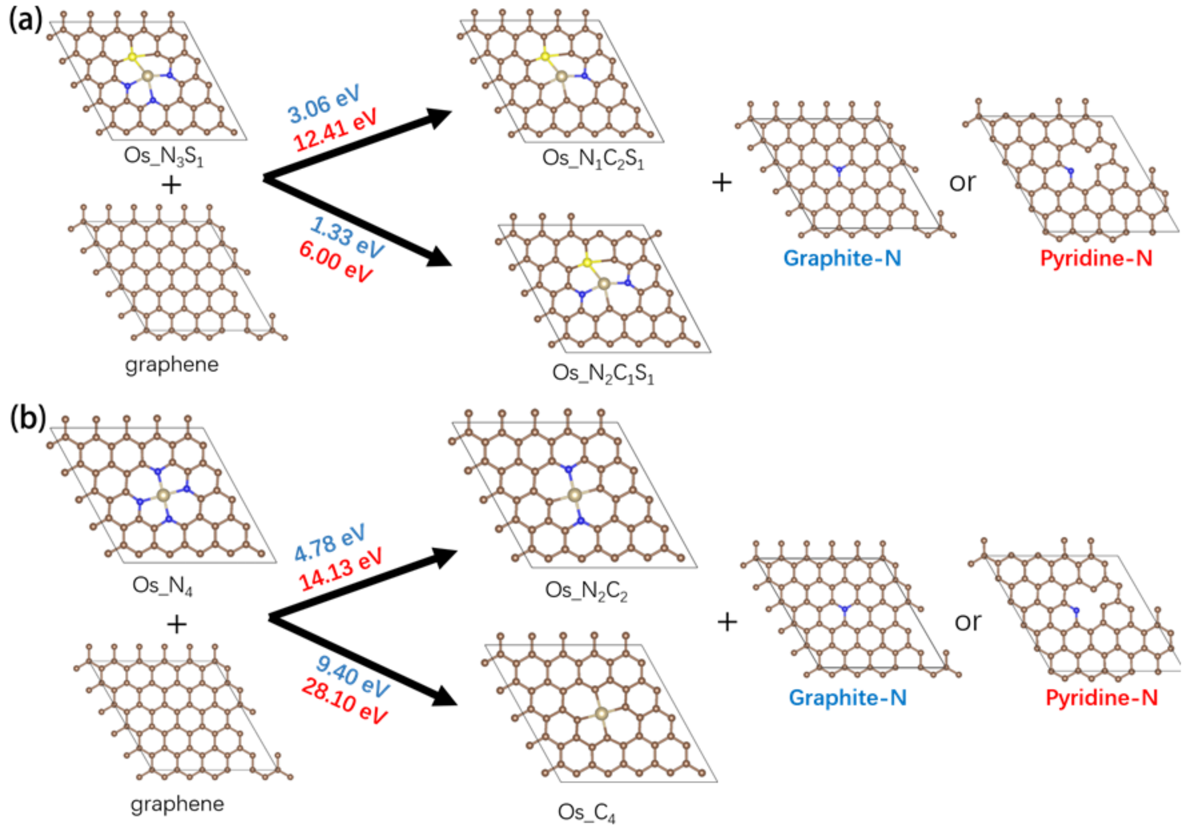

Supplementary Figure 29. Bonding thermodynamics priority between Os-N and Os-C. Energy change of the process from coordination configuration with Os-N bonds to that with Os-C bonds in **a** Os/CNS and **b** Os/CN.

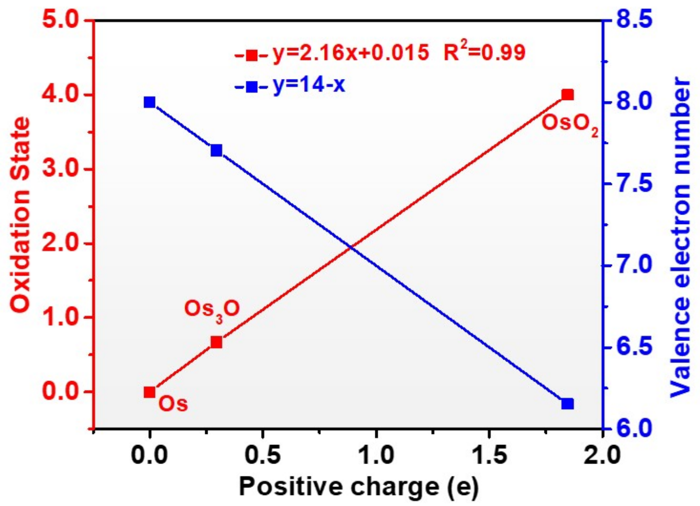

Supplementary Figure 30. Calculation of theoretical oxidation state. The scaling relationship among valence electron number, oxidation state, and positive charge of Os element.

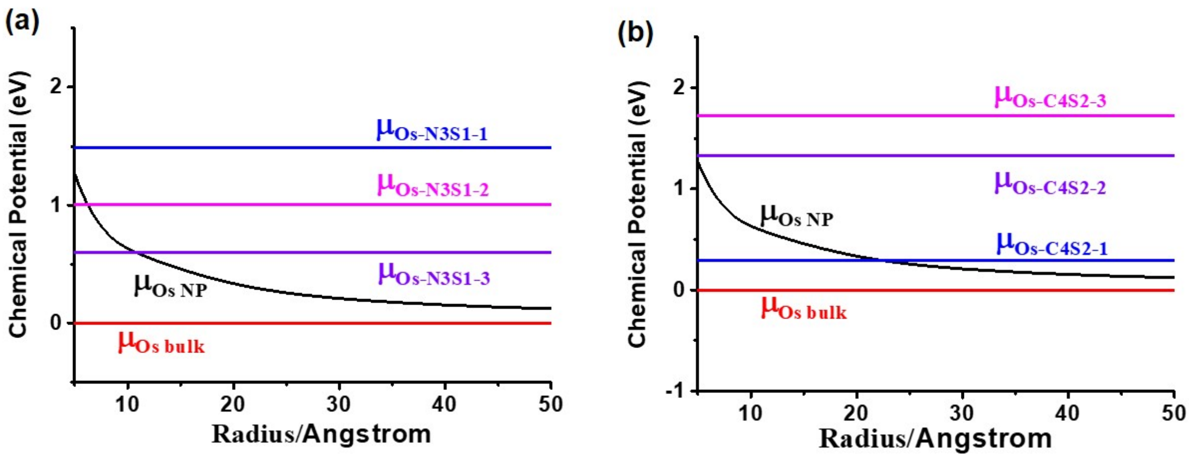

Supplementary Figure 31. Anti-aggregation assessment for Os SACs with diverse coordination. Comparison between chemical potential of Os nanoparticle, μ_{NP}(R), with respect to curvature of NP, that of Os metal bulk, μ_{Os} bulk, and those of alternative configuration of **a** Os-N_3S_1 and **b** Os-C_4S_2.

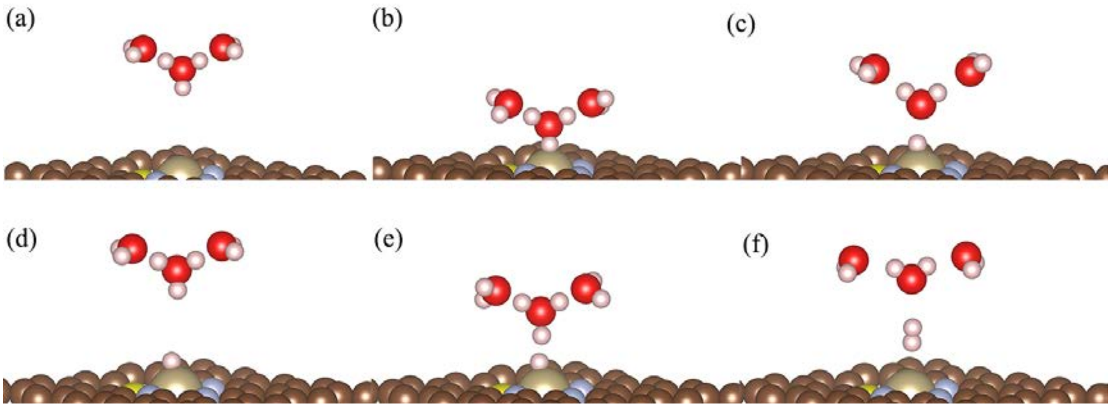

Supplementary Figure 32. The configurations of different mechanism on Os-N_3S_1. The configuration of **a** initial state, **b** transition state and **c** final state for Volmer mechanism on Os-N_3S_1. The configuration of **d** initial state, **e** transition state and **f** final state for Heyrovsky mechanism on Os-N_3S_1.

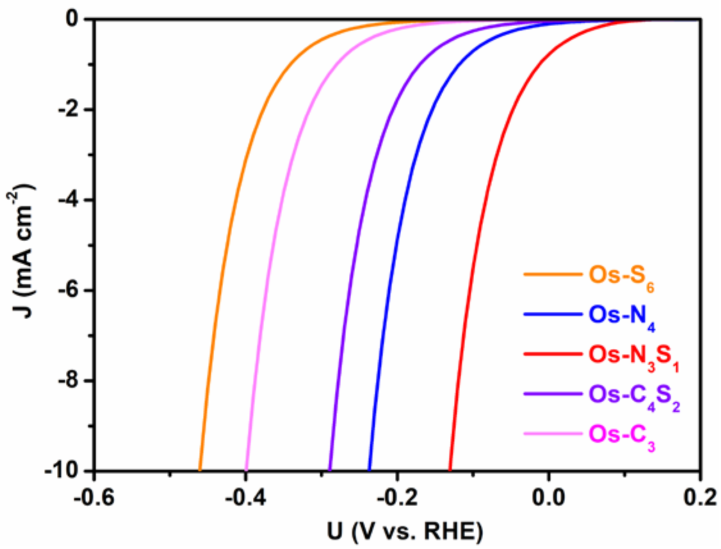

Supplementary Figure 33. The calculated LSV curves. The polarization curve simulation for different Os SACs based on microkinetics model.

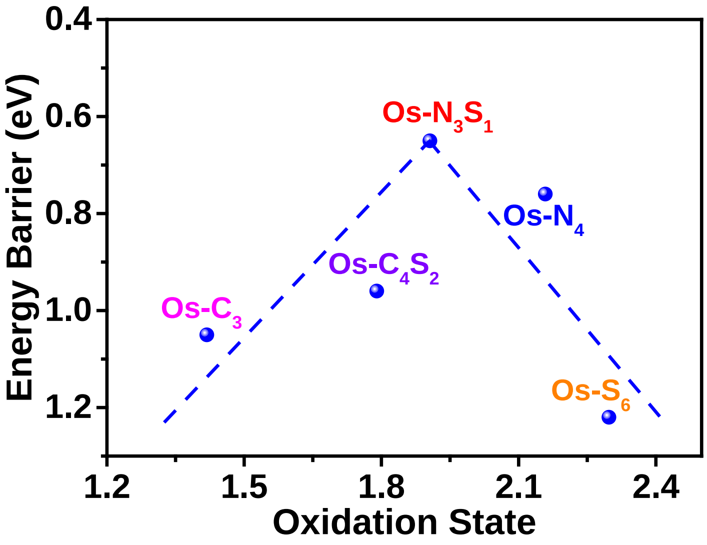

Supplementary Figure 34. The relationship between oxidation state and energy barrier. Energy barrier as a function of oxidation state for different Os SACs.

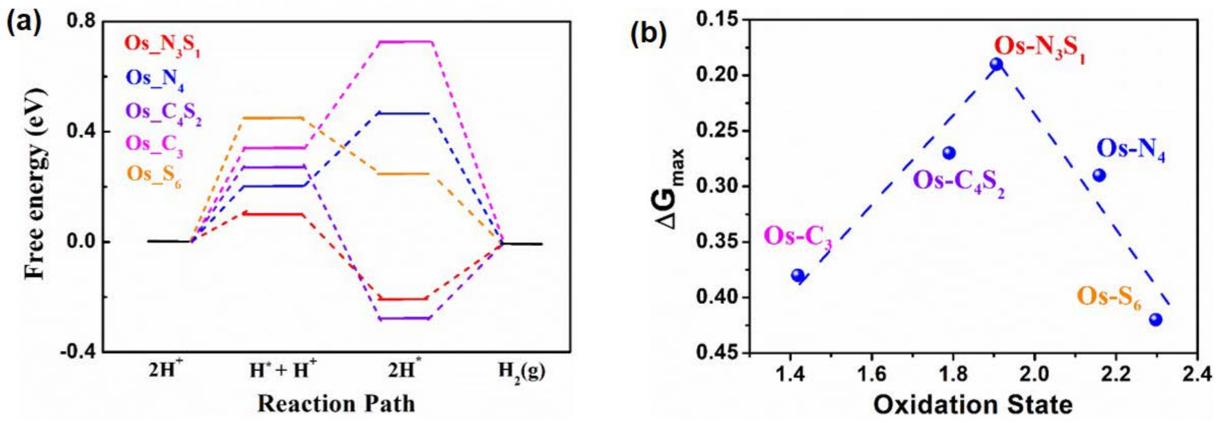

Supplementary Figure 35. The DFT calculation of Volmer-Tafel mechanism. **a** Gibbs free energy diagram of HER in acid media through Volmer-Tafel mechanism on Os SACs. **b** The free energy change of potential-determining step as a function of oxidation state for different Os SACs.

Supplementary Table 1. EXAFS fitting parameters at the Os L_{III}-edge for various samples.

Sample	Shell	N [a]	R (Å) [b]	R factor (%)
Os/CN	Os-N	4.4	2.07	0.4
Os/CNS	Os-N	3.1	2.07	1.1
	Os-S	0.9	2.36	
Os/CS	Os-C	3.7	2.04	0.4
	Os-S	2.6	2.31	
Os/CS-2	Os-S	5.8	2.30	0.1
Os/C	Os-C	3.2	1.94	1.1
	Os-C*	12.9	2.51	
Os NPs	Os-Os	8.2	2.69	1.5

Note: the precision range of R is ± 0.03 Å.

***** Second nearest neighbor coordination.

Supplementary Table 2. Comparison of the HER performance for different catalysts.

Electrocatalysts	Overpotential at 10 mA cm^{-2} (mV)	Tafel slope (mV dec^{-1})	TOF (s^{-1})	Solution	References
Pt/A-NiCo LDH	16	38.8	—	1 M KOH	1
Cu_{sub}@MoS_2	160	86	8.27 at 400 mV	0.5 M H_2SO_4	2
Pt-SAs/CoNC	57	64.5	—	0.5 M H_2SO_4	3
Pt_1/OLC	38	35	40. 78 at 100 mV	0.5 M H_2SO_4	4
Pt SASs/AG	12	29.33	—	0.5 M H_2SO_4	5
Pt_1/NMC	~30	26	—	0.5 M H_2SO_4	6
Os-$OsSe_2$	26	31	—	0.5 M H_2SO_4	7
B-Os aerogels	12	26.8	2.5 at 50 mV	0.5 M H_2SO_4	8
Ir-doped WO_3	36	72	—	0.5 M H_2SO_4	9
W-SA	85	53	3.40 at 80 mV	0.1 M KOH	10
Pt_{SA}/α-MoC_{1-x}@C	12	27	31.98 at 100 mV	0.5 M H_2SO_4	11
IrNi-N-C	28	38	3.06 at 50 mV	0.5 M H_2SO_4	12
Ru_{SA+NP}/DC	16.6	28.7	0.27 at 30 mV	0.5 M H_2SO_4	13
Pt_1/NMHCS	40	56	20.18 at 300 mV	0.5 M H_2SO_4	14
0.1Pt doped MoO_3	~26	~33.2	11.5 at 100 mV	0.5 M H_2SO_4	15
Os/CNS	22	41	10.55 at 50 mV	0.5 M H_2SO_4	This work

Supplementary Table 3. The oxidation state of Os atom and bond length of Os-neighbor atom for each possible active center of different Os SACs shown in Figure S28.

Os SACs	Oxidation state of Os Atom	Bond Length of Os-neighbor atom (Å)
**Os-S_6**	2.297	2.35x6 (Os-S)
**Os-N_4**	2.159	1.95x4 (Os -N)
**Os-N_3S_1-1**	2.026	1.98x3(Os -N), 2.16(Os -S)
**Os-N_3S_1-2**	1.850	1.97x3(Os -N), 2.24(Os -S)
**Os-N_3S_1-3**	1.906	1.98x3(Os -N), 2.37(Os -S)
**Os-C_4S_2-1**	1.790	2.09x4(Os -C), 2.38x2 (Os -S)
**Os-C_4S_2-2**	1.392	2.26x4(Os -C), 2.33x2 (Os -S)
**Os-C_4S_2-3**	1.327	2.31x4(Os -C), 2.26x2 (Os -S)
**Os-C_4S_2-4**	1.652	2.01x4(Os -C), 2.77x2 (Os -S)
**Os-C_4S_2-5**	3.081	1.89x4(Os -C), 2.65x2 (Os -S)
**Os-C_3**	1.418	1.88x3(Os-C)

Supplementary Table 4. Values used for the entropy and zero-point energy corrections in determining the free energy of free H_2 and H_2O molecule.

**Species**	**T×S (eV) (298K)**	**ZPE (eV)**
H_2	0.41	0.27
H_2O(0.035 bar)	0.58	0.56

Supplementary Table 5. DFT adsorption energies (ΔE_{ad}, eV), zero-point energy (ZPE, eV), zero-point energy correction (ΔZPE, eV), entropy (T*S, eV, T=298K), entropy correction (T*ΔS, eV, T=298K), adsorption free energies (ΔG_{ad}, eV) of the adsorbed H along reaction pathway of acidic HER on Os SACs. Adsorption energies (eV) were taken relative to energies of free H_2 and H_2O molecule.

Os SACs	$\mathbf{\Delta E_{ad}H}$	$\mathbf{ZPE_H}$	$\mathbf{\Delta ZPE_H}$	$\mathbf{T{*}S_H}$	$\mathbf{T{*}\Delta S_H}$	$\mathbf{\Delta G_{ad}H}$
Os-S_6	0.14	0.22	0.08	0.00	-0.20	0.42
Os-N_4	-0.09	0.23	0.09	0.00	-0.20	0.20
Os-N_3S_1	-0.19	0.22	0.08	0.02	-0.18	0.07
Os-C_4S_2	-0.01	0.21	0.07	0.01	-0.19	0.25
Os-C_3	0.10	0.21	0.07	0.01	-0.19	0.36

Supplementary Table 6. Electron occupation in *d*-orbital under Fermi energy derived from density of state analysis in Fig. 5a.

Os-S_6	Os-N_4	Os-N_3S_1	Os-C_4S_2	Os-C_3
1.47	1.85	2.17	2.77	2.96

Supplementary References

1. Fan, B. et al. Single atoms (Pt, Ir and Rh) anchored on activated NiCo LDH for alkaline hydrogen evolution reaction. Chem. Commun. 58, 8254-8257 (2022).
2. Li, Z. et al. Manipulating coordination structures of mixed-valence copper single atoms on 1T-MoS_2 for efficient hydrogen evolution. ACS Catal. 12, 7687-7695 (2022).
3. Zhao, Y. et al. Modulating Pt-O-Pt atomic clusters with isolated cobalt atoms for enhanced hydrogen evolution catalysis. Nat. Commun. 13, 2430 (2022).
4. Liu, D. et al. Atomically dispersed platinum supported on curved carbon supports for efficient electrocatalytic hydrogen evolution. Nat. Energy 4, 512-518 (2019).
5. Ye, S. et al. Highly stable single Pt atomic sites anchored on aniline-stacked graphene for hydrogen evolution reaction. Energy Environ. Sci. 12, 1000-1007 (2019).
6. Wei, H. et al. Ultralow-temperature photochemical synthesis of atomically dispersed Pt catalysts for the hydrogen evolution reaction. Chem. Sci. 10, 2830-2836 (2019).
7. Chen, D. et al. Work-function-induced interfacial built-in electric fields in Os-$OsSe_2$ heterostructures for active acidic and alkaline hydrogen evolution. Angew Chem. Int. Ed. e202208642 (2022).
8. Li, Y. et al. Interstitial boron-triggered electron-deficient Os aerogels for enhanced pH-universal hydrogen evolution. Nat. Commun. 13, 1143 (2022).
9. Li, P. et al. Iridium in tungsten trioxide matrix as an efficient Bi-functional electrocatalyst for overall water splitting in acidic media. Small 17, 2102078 (2021).
10. Chen, W. et al. Single tungsten atoms supported on MOF-derived N-doped carbon for robust electrochemical hydrogen evolution. Adv. Mater. 30, 1800396 (2018).
11. Wang, W. et al. Confining zero-valent platinum single atoms in α-MoC_{1-x} for pH-universal hydrogen evolution reaction. Adv. Funct. Mater. 32, 2108464 (2022).
12. Liu, D. et al. Triggering electronic coupling between neighboring hetero-diatomic metal sites promotes hydrogen evolution reaction kinetics. Nano Energy 98, 107296 (2022).
13. Zhang, L. et al. Exploring the dominant role of atomic- and nano-ruthenium as active sites for hydrogen evolution reaction in both acidic and alkaline Media. Adv. Sci. 8, 2004516 (2021).
14. Kuang, P. et al. Pt single atoms supported on N-doped mesoporous hollow carbon spheres with enhanced electrocatalytic H_2-evolution activity. Adv. Mater. 33, 2008599 (2021).
15. Zhu, Y. et al. Atomically dispersed Pt-O coordination boosts highly active and durable

acidic hydrogen evolution reaction. Chem. Eng. J. 440, 135957 (2022).
